# Short-term efficacy of repetitive transcranial magnetic stimulation (rTMS) in depression- reanalysis of data from meta-analyses up to 2010

**DOI:** 10.1186/s40359-014-0039-y

**Published:** 2014-10-07

**Authors:** Karina Karolina Kedzior, Sarah Kim Reitz

**Affiliations:** Bremen International Graduate School of Social Sciences (BIGSSS), Jacobs University Bremen, Campus Ring 1, 28759 Bremen, Germany

**Keywords:** Major depression, Meta-analysis, Randomised-controlled trial (RCT), High-frequency rTMS, Systematic review

## Abstract

**Background:**

According to a narrative review of 13 meta-analyses (published up to 2010), repetitive transcranial magnetic stimulation (rTMS) has a moderate, short-term antidepressant effect in the treatment of major depression. The aim of the current study was to reanalyse the data from these 13 meta-analyses with a uniform meta-analytical procedure and to investigate predictors of such an antidepressant response.

**Methods:**

A total of 40 double-blind, randomised, sham-controlled trials with parallel designs, utilising rTMS of the dorsolateral prefrontal cortex in the treatment of major depression, was included in the current meta-analysis. The studies were conducted in 15 countries on 1583 patients and published between 1997–2008. Depression severity was measured using the Hamilton Depression Rating Scale, Beck Depression Inventory, or Montgomery Åsberg Depression Rating Scale at baseline and after the last rTMS. A random-effects model with the inverse-variance weights was used to compute the overall mean weighted effect size, Cohen’s *d*.

**Results:**

There was a significant and moderate reduction in depression scores from baseline to final, favouring rTMS over sham (overall *d* = −.54, *95% CI: −*.68, −.41, *N* = 40 studies). Predictors of such a response were investigated in the largest group of studies (*N* = 32) with high-frequency (>1 Hz) left (HFL) rTMS. The antidepressant effect of HFL rTMS was present univariately in studies with patients receiving antidepressants (at stable doses or started concurrently with rTMS), with treatment-resistance, and with unipolar (or bipolar) depression without psychotic features. Univariate meta-regressions showed that depression scores were significantly lower after HFL rTMS in studies with higher proportion of female patients. There was little evidence for publication bias in the current analysis.

**Conclusions:**

Daily rTMS (with any parameters) has a moderate, short-term antidepressant effect in studies published up to 2008. The clinical efficacy of HFL rTMS may be better in female patients not controlling for any other study parameters.

**Electronic supplementary material:**

The online version of this article (doi:10.1186/s40359-014-0039-y) contains supplementary material, which is available to authorized users.

## Background

Repetitive transcranial magnetic stimulation (rTMS) is an effective treatment against medication-resistant unipolar depression. According to a narrative review of 13 meta-analyses (published between 2001–2010), the clinically-meaningful effect of daily rTMS of the dorsolateral prefrontal cortex (DLPFC) was observed in double-blind, randomised-controlled trials (RCTs) with inactive sham groups, published between 1995–2008 (Dell’Osso et al. 2011). According to these meta-analyses, such an effect was investigated mostly in the short-term (baseline to last rTMS session) treatment of major depression, during the double-blind phases of RCTs.

Regardless of such a high interest in this topic, the antidepressant effect of rTMS was found to be moderate and rTMS parameters of clinical relevance were only partially established in the past 13 meta-analyses (Dell’Osso et al. 2011). The past meta-analyses showed that the short-term antidepressant effect was most consistently observed in the largest subgroup of RCTs using the high frequency (>1 Hz) left (HFL) stimulation of the DLPFC (Dell’Osso et al. 2011). In addition, only very few meta-analyses (based on a small number of RCTs) showed that the low frequency (≤1 Hz) right (LFR) rTMS and bilateral (or sequential) rTMS also appear to have antidepressant properties in the short-term (Herrmann and Ebmeier 2006; Schutter 2010; Slotema et al. 2010). Regardless of frequency/location, the antidepressant effect of rTMS occurred after 10 or 15 sessions of treatment (Gross et al. 2007; Martin et al. 2003; Rodriguez-Martin et al. 2001). However, there was no association between the antidepressant effect and the duration of treatment nor any other rTMS parameters, such as the frequency of stimulation, resting motor threshold, stimuli/session, or total stimuli/study (Herrmann and Ebmeier 2006; Holtzheimer et al. 2001; Schutter 2009; Slotema et al. 2010).

Similarly to rTMS parameters, the demographic and clinical predictors of rTMS response were not consistently established in the past 13 meta-analyses (Dell’Osso et al. 2011). For example, effect sizes were unrelated to the mean age of patients (Herrmann and Ebmeier 2006). Furthermore, rTMS was effective as a monotherapy, in studies with patients on concurrent antidepressants (Burt et al. 2002; Herrmann and Ebmeier 2006; Slotema et al. 2010), and in studies with treatment-resistant patients (Herrmann and Ebmeier 2006; Lam et al. 2008; Schutter 2009). The authors of some meta-analyses suggested that the antidepressant effect of rTMS could be enhanced in less severely resistant patients (Gross et al. 2007; Holtzheimer et al. 2001). Finally, the antidepressant effect of rTMS was observed in studies with unipolar and bipolar patients (Dell’Osso et al. 2011) and non-psychotic patients (Slotema et al. 2010).

It is not surprising that consistent outcomes were not observed considering the heterogeneous aims and approaches to meta-analysis utilised in the past 13 meta-analyses up to 2010. In general, all 13 meta-analyses were published before the Preferred Reporting Items for Systematic Reviews and Meta-Analyses (PRISMA) guidelines were established (Moher et al. 2009). These guidelines were established to improve the quality of systematic reviews in terms of consistent reporting of all steps of such reviews, including the literature search procedures, study selection, assessment of publication bias, description of statistical details of the analyses, and presenting of results (Moher et al. 2009) and have been implemented in the newest meta-analyses on this topic published after 2010 (for review see Kedzior et al. 2014). Our inspection of the 13 meta-analyses up to 2010 revealed that, although similar databases, search terms, and timeframes were used, the analyses included a different number of primary studies published between 1995–2008 (for more details see Additional file 1). Some overlap in the primary studies suggests that similar inclusion and exclusion criteria were applied although specific aims differed among the 13 meta-analyses. Furthermore, except for one study (Holtzheimer et al. 2001), the statistical approach was not adequately described in the 13 meta-analyses. It was especially unclear how baseline depression scores were controlled for when computing effect sizes in most of the 13 meta-analyses. Since many studies utilised different rTMS parameters in multiple subgroups of patients (with only one sham group/study), multiple depression scales, and multiple points in time (baseline and final), the statistical approach to reducing such complex data sets to single effect sizes/study should be adequately explained to better understand the reliability of results. Based on the random selection of all available studies on this topic, the correct (more statistically conservative) random-effects model of meta-analysis was applied in most of the 13 meta-analyses. However, the weighting method of effect sizes was often not explicitly explained. Since studies with positive and significant effect sizes are more likely to be submitted for peer-review and published (Borenstein et al. 2009), a resulting publication bias was assessed, although inconsistently (using different tests), in the 13 meta-analyses. Finally, since too few homogenous studies were available for moderator analyses (subgroup analyses or meta-regressions), such analyses were either not conducted at all or, if conducted, the statistical power to detect any significant predictors was often low.

Therefore, the aim of the current study was to apply a uniform and transparent (explicitly described) meta-analytical procedure to reanalyse the data from the past 13 meta-analyses (published until 2010 and conducted using heterogeneous statistical methods). Although such a reanalysis could be considered a replication rather than a novel study, replications are necessary in science to more reliably confirm or synthesise the findings of others (Laws 2013). In particular, our aim was to find out if the reanalysis of data from the primary studies published until 2008 with one method of meta-analysis would produce only a moderate short-term antidepressant effect of rTMS (like the one observed in most of the past 13 meta-analyses) or if the effect would increase due to a uniform statistical approach used in this overall meta-analysis. It was also of interest to test if the inclusion of more data than any one of the past meta-analyses alone would allow us to detect any significant predictors of the short-term response to rTMS due to a higher statistical power of such an overall analysis. The choice of predictors was based on the data presented in the past 13 meta-analyses and included clinical and demographic characteristics of patients and parameters of rTMS. In addition, we have included gender (measured as percentage of female patients/study) as another predictor because none of the past 13 meta-analyses investigated the relationship between gender and the response to rTMS although depression is more prevalent among females than males worldwide (Bromet et al. 2011). The update of the current meta-analysis using data from primary RCTs identified in a novel systematic literature search and published after 2008 was published recently (Kedzior et al. 2014).

It was hypothesised that, when controlling for baseline, a significant antidepressant effect favouring rTMS over sham would be observed in HFL, LFR, and bilateral/sequential studies based on the findings from the past 13 meta-analyses. If statistical heterogeneity alone were to blame for relatively low effect sizes in the 13 meta-analyses then it was expected that the effect sizes would be higher utilising one uniform method of meta-analysis in the current study. Finally, we expected to find significant predictors of antidepressant response to rTMS (patient characteristics and/or rTMS parameters) due to the improved statistical power resulting from the highest number of studies included in the current compared to the past meta-analyses.

## Methods

The PRISMA checklist listing the precise location of various steps of this meta-analysis is included in the Additional file 1.

### Study Selection

The primary studies used in the current meta-analysis were selected from the past 13 meta-analyses published between 2001–2010 (Dell’Osso et al. 2011). The details of the systematic literature search strategy used in each of these 13 meta-analyses are summarised in the Additional file 1: Table S1. Most past meta-analyses utilised Medline or PubMed databases and similar search terms including ‘depression’ and ‘rTMS’.

Various combinations of *N* = 53 primary sources published between 1995–2008 were included in the past 13 meta-analyses (see the Additional file 1: Table S2). The study selection procedure and exclusion criteria used in the current meta-analysis are summarised in the PRISMA flowchart (Moher et al. 2009), Figure 1. Studies were excluded mostly because inadequate data were reported to compute the effect sizes and the authors failed to reply to email requests and/or provide additional data. The final meta-analysis was performed on the data from 40 out of 53 studies which met the following inclusion criteria:

**Figure 1:**
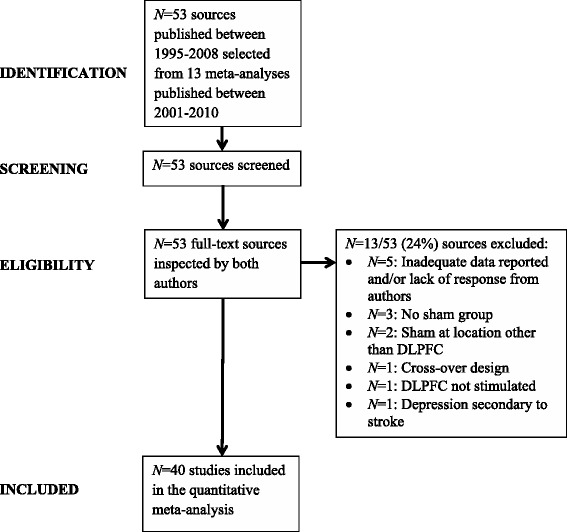
**Study selection and exclusion criteria.** Note: Abbreviations: DLPFC, dorsolateral prefrontal cortex; *N*, number of sources.

- double-blind RCT with an inactive sham group,
- parallel design (cross-over designs might produce data biased by carry-over effects and thus such data were excluded from the current analysis),
- active rTMS (with any frequency of stimulation) and sham administered at the same DLPFC location (left, right, bilateral or sequential),
- patients with primary diagnoses of major depressive episode or disorder according to DSM-IV and/or ICD-10 criteria (unipolar or bipolar, non-psychotic or psychotic),
- depression measured at baseline and on the last session of rTMS or sham during the double-blind phase of a study,
- depression measured according to any version of Hamilton Depression Rating Scale, HAMD (Hamilton 1960), Beck Depression Inventory, BDI (Beck et al. 1961), or Montgomery Åsberg Depression Rating Scale, MADRS (Montgomery and Asberg 1979),
- adequate data provided to compute effect sizes or author contact details available for additional data requests.

### Data extraction

Data were extracted from all *N* = 40 RCTs by both authors independently and any inconsistencies were resolved between the authors via consensus. In some cases depression scores were extrapolated from figures (using physical measurements of the printed figures) by both authors independently and a mean of both estimations was used in the final analyses. The extracted data were also cross-checked against the data shown in the past 13 meta-analyses. The rTMS parameters, clinical characteristics of patients, and mean depression scores (baseline and final in rTMS and sham groups) are shown in Tables 1 and 2 respectively.

**Table 1:** **rTMS parameters in the** ***N*** **= 40 RCTs included in the current meta-analysis**

**Study (by year and first author); country**	**DLPFC location**	**Definition of location**	**Frequency (Hz)**	**Motor threshold (%)**	**Coil type**	**Coil diameter (mm)**	**Coil angle sham (°)**	**Stimuli/session**	**Trains/session**	**Inter-train interval (s)**	**Sessions**	**Stimulator (company)**
George et al. (1997); USA^a^	L	5 cm	20	80	F8	–	45	800	–	58	10	Cadwell
Avery et al. (1999); USA	L	5 cm	10	80	–	–	45	–	20	55	10	Cadwell
Kimbrell et al. (1999); USA^a^	L	5 cm	20	80	F8	–	45	800	20	60	10	Cadwell
	L		1									
Klein et al. (1999); Israel	R	6 cm	1	110	C	90	90	120	–	180	10	Cadwell
Loo et al. (1999); Australia	L	5 cm	10	110	F8	70	45	–	30	30	10	MagStim
Padberg et al. (1999); Germany	L	5 cm	10	90	F8	70	90	250	5	30	5	MagStim
	L		.3						10			
Berman et al. (2000); USA	L	5 cm	20	80	F8	–	45	–	20	58	10	Cadwell
Eschweiler et al. (2000); Germany^a,b^	L	5 cm	10	90	F8	70	90	–	20	50	5	MagStim
George et al. (2000); USA^c^	L	5 cm	12*	100	F8	–	45	1600	–	25*	10	Cadwell
Garcia-Toro et al. (2001a); Spain	L	5 cm	20	90	F8	–	90	–	30	30*	10	MagPro
Garcia-Toro et al. (2001b); Spain	L	5 cm	20	90	F8	85	90	1200	30	30*	10	MagPro
Manes et al. (2001) ; USA	L	MRI	20	80	F8	–	TI	–	20	60	5	MagStim
Boutros et al. (2002); USA	L	5 cm	20	80	F8	70	90	800	20	58	10	MagStim
Padberg et al. (2002); Germany^d^	L	5 cm	10	100	F8	70	90	1500	15	30	10	MagStim
Fitzgerald et al. (2003); Australia	L	5 cm	10	100	F8	70	45	1000	20	25	10	MagStim
	R		1					300	5	60		
Höppner et al. (2003); Germany^e^	L	5 cm	20	90	F8	–	90	–	20	60	10	MagLite
Loo et al. (2003); Australia	B	5 cm	15	90	F8	70	TI	–	24	25	15	MagStim
Nahas et al. (2003); USA	L	5 cm	5	110	F8	–	45	1600	–	22	10	Neotonus
Buchholtz et al. (2004); Denmark	L	5 cm	10	90	F8	70	90	–	20	60	15	MagStim
Hausmann et al. (2004); Austria^f^	BS	MRI	11*	110*	F8	–	TI	2300*	10	10	10	MagStim
Holtzheimer et al. (2004); USA	L	5 cm	10	110	F8	–	45	1600	32	45*	10	MagPro
Kauffmann et al. (2004); USA	R	5 cm	1	110	C	90	45	120	2	180	10	MagLite
Koerselman et al. (2004); Netherlands	L	5 cm	20	80	C	60	45	–	20	30	10	MagPro
Mosimann et al. (2004); Switzerland	L	5 cm	20	100	F8	–	90	1600	40	28	10	MagStim
Poulet et al. (2004); France	L	5 cm	10	80	F8	70	45	–	20	58	10	MagStim
Rossini et al. (2005); Italy	L	5 cm	15	100	F8	70	90	900	30	28	10	MagStim
Rumi et al. (2005); Brazil	L	5 cm	5	120	F8	–	TS	1250	25	20	20	MagPro
Su et al. (2005); Taiwan^c^	L	5 cm	12*	100	F8	70	90	–	40	–	10	MagStim
Avery et al. (2006); USA	L	MRI	10	110	F8	70	90	1600	32	28*	15	MagPro
Fitzgerald et al. (2006); Australia	BS	5 cm	6*	105*	F8	70	45	–	18	28*	10	MagPro
Garcia-Toro et al. (2006); Spain^g^	BS	5 cm	11*	110	F8	85	45	–	60	20*	10	MagPro
Januel et al. (2006); France	R	5 cm	1	90	F8	–	TS	–	2	180	16	MagStim
Anderson et al. (2007); UK	L	5 cm	10	110	F8	–	TS	1000	20	30	12	MagStim
Bortolomasi et al. (2007); Italy	L	5 cm	20	90	C	90	90	800	20	60	5	MagStim
Herwig et al. (2007); Germany/Austria^h^	L	F3	10	110	F8	70	45	2000	100	8	15	MagStim/Pro
Loo et al. (2007); Australia^i^	L	5 cm	10	110	F8	70	TI	–	30	25	20	MagStim
O’Reardon et al. (2007); USA, Australia, Canada	L	5 cm	10	120	–	–	TS	3000	–	26	20	Neuronetics
Stern et al. (2007); USA	L	5 cm	10	110	F8	–	90	–	20	52	10	MagStim/MagPro
	L		1						1	–		
	R		1						1	–		
Bretlau et al. (2008); Denmark	L	5 cm	8	90	F8	70	TS	1289	20	52	15	MagStim
Mogg et al. (2008); UK	L	5 cm	10	110	F8	–	TS	1000	20	55	10	MagStim

Notes: *Mean values. Sham was always administered at the same DLPFC location (left or right or bilateral) as the active rTMS (for the definition of DLPFC location, ‘5 cm’ refers to 5 cm rostral (anterior) to sagittal (parasagittal) plane). ^a^If cross-over design was used then the results of only the parallel double-blind stimulation are included in the current analysis. ^b^The combined scores of two active rTMS groups (group 1 and 3) are included in the current analysis. ^c^The combined scores of two HFL rTMS groups (5 Hz and 20 Hz) are included in the current analysis. ^d^Since sham was administered at 100% MT, only the active rTMS with 100% MT group is included in the current analysis. ^e^Since sham was administered to the left DLPFC, only the HFL rTMS group is included in the current analysis. ^f^The ‘active rTMS’ group consists of group A1 (HFL rTMS followed by right-sham) and group A2 (HFL rTMS followed by LFR rTMS). Sham was applied bilaterally (left then right DLPFC). ^g^’Active TMS’ group is included in the current analysis (active TMS after single photon emission computed tomography, SPECT, is excluded because patients in this group received rTMS at individualised sites based on the results of SPECT). ^h^Sham was administered 5 cm lateral to the F3 location, above the left temporal muscle. ^i^In contrast to all other studies that utilised a single rTMS (or sham) session/day, rTMS was applied twice/day for 2 weeks, 5 days/week (thus a total of 20 sessions). Abbreviations: B, bilateral DLPFC; BS, bilateral sequential (left then right DLPFC); C, circular; DLPFC, dorsolateral prefrontal cortex; F3, the F3 location of the 10–20 electroencephalogram (EEG) system; F8, figure-of-eight shape; L, left DLPFC; MRI, magnetic resonance imaging; R, right DLPFC; RCT, randomised-controlled trial; rTMS, repetitive transcranial magnetic stimulation; TI, tangential with inactive coil; TS, tangential with sham coil containing embedded magnetic shield.

**Table 2:** **Patient characteristics at baseline and depression scores in the active rTMS and sham groups in** ***N*** **= 40 RCTs**

**Study**	**Mean age (all patients)**	**Female (% all patients)**	**Treatment-resistance** ^**A**^	**Bipolar (%)** ^**B**^	**Psychotic (%)** ^**C**^	**Medication** ^**D**^	**Data source**	**Scale** ^**E**^	**Mean ±** ***SD*** **(number of patients) depression severity score**					
									**Baseline**		**Last session** ^**F**^		**Baseline – last session**	
									**Sham**	**rTMS**	**Sham**	**rTMS**	**Sham**	**rTMS**
George et al. (1997)	42	92%	some	+ 8%	N/A	+	Tab one	HAMD21	26 ± 3 (5)	30 ± 4 (7)	30 ± 8 (5)	23 ± 9 (7)	−4 ± 7 (5)	7 ± 8 (7)
Avery et al. (1999)	44	83%	+	+ 17%	–	+	Tab one	HAMD21	20 ± 8 (2)	21 ± 7 (4)	15 ± 2 (2)	11 ± 4 (4)	5 ± 7 (2)	10 ± 6 (4)
								BDI21	20 ± 7 (2)	28 ± 7 (4)	14 ± 11 (2)	20 ± 11 (4)	6 ± 10 (2)	8 ± 10 (4)
Kimbrell et al. (1999)	42	54%	N/A	+ 31%	N/A	–	All (Tab one)	HAMD21	24 ± 7 (3)	30 ± 8 (10)	25 ± 10 (3)	27 ± 10 (10)	−1 ± 9 (3)	3 ± 9 (10)
							20Hz		24 ± 7 (3)	25 ± 7 (5)	25 ± 10 (3)	28 ± 8 (5)	−1 ± 9 (3)	−3 ± 8 (5)
							1Hz		24 ± 7 (3)	34 ± 8 (5)	25 ± 10 (3)	27 ± 13 (5)	−1 ± 9 (3)	7 ± 11 (5)
Klein et al. (1999)	59	76%	–	+ 19%	+ 23%	+	Tab two	HAMD17	25 ± 6 (32)	26 ± 6 (35)	20 ± 10 (32)	14 ± 9 (35)	5 ± 9 (32)	12 ± 8 (35)
								MADRS	34 ± 8 (32)	34 ± 5 (35)	27 ± 12 (32)	20 ± 12 (35)	7 ± 11 (32)	14 ± 10 (35)
Loo et al. (1999)	48	50%	some	+ 17%	+ 6%	+	Authors	HAMD21	25 ± 6 (9)	21 ± 7 (9)	19 ± 7 (9)	17 ± 7 (9)	6 ± 7 (9)	4 ± 7 (9)
								MADRS	38 ± 6 (9)	33 ± 9 (9)	29 ± 10 (9)	26 ± 9 (9)	9 ± 9 (9)	7 ± 9 (9)
Padberg et al. (1999)	51	61%	+	–	N/A	+	All	HAMD21	22 ± 9 (6)	28 ± 9 (12)	24 ± 10 (6)	25 ± 8 (12)	−2 ± 10 (6)	3 ± 8 (12)
							Text 10Hz		22 ± 9 (6)	30 ± 10 (6)	24 ± 10 (6)	28 ± 9 (6)	−2 ± 10 (6)	2 ± 10 (6)
							Fig one 0.3Hz		22 ± 9 (6)	27 ± 9 (6)	24 ± 10 (6)	22 ± 6 (6)	−2 ± 10 (6)	5 ± 8 (6)
Berman et al. (2000)	42	30%	some	+ 5%	+ 5%	–	Tab one	HAMD25	37 ± 8 (10)	37 ± 10 (10)	36 ± 9 (10)	25 ± 9 (10)	1 ± 8 (10)	12 ± 10 (10)
Eschweiler et al. (2000)	57	67%	N/A	–	+ 8%	+	Tab one	HAMD21	20 ± 4 (5)	27 ± 4 (7)	23 ± 6 (5)	22 ± 5 (7)	−3 ± 5 (5)	5 ± 5 (7)
								BDI21	28 ± 10 (5)	40 ± 6 (7)	32 ± 9 (5)	33 ± 11 (7)	−4 ± 10 (5)	7 ± 10 (7)
George et al. (2000)	44	63%	N/A	+ 30%	–	–	All (Tab one)	HAMD21	24 ± 4 (10)	28 ± 6 (20)	19 ± 6 (10)	18 ± 9 (20)	5 ± 5 (10)	10 ± 8 (20)
Garcia-Toro et al. (2001a)	51	43%	+	–	N/A	+	Tab two	HAMD21	26 ± 5 (18)	27 ± 7 (17)	24 ± 4 (18)	20 ± 6 (17)	2 ± 5 (18)	7 ± 7 (17)
								BDI17	26 ± 6 (18)	27 ± 9 (17)	24 ± 5 (18)	22 ± 7 (17)	2 ± 6 (18)	5 ± 8 (17)
Garcia-Toro et al. (2001b)	44	55%	–	N/A	N/A	+D1	Tab one	HAMD21	27 ± 6 (11)	26 ± 6 (11)	18 ± 9 (11)	16 ± 8 (11)	9 ± 8 (11)	10 ± 7 (11)
								BDI17	23 ± 7 (11)	27 ± 8 (11)	21 ± 8 (11)	19 ± 7 (11)	2 ± 8 (11)	8 ± 8 (11)
Manes et al. (2001)	61	50%	some	N/A	N/A	–	Tab two	HAMD21	23 ± 7 (10)	23 ± 5 (10)	16 ± 8 (10)	14 ± 5 (10)	7 ± 8 (10)	9 ± 5 (10)
Boutros et al. (2002)	51	22%	+	–	N/A	+	Tab two	HAMD25	36 ± 4 (7)	40 ± 10 (11)	26 ± 13 (7)	27 ± 13 (11)	10 ± 12 (7)	13 ± 12 (11)
Padberg et al. (2002)	57	70%	+	N/A	N/A	+	All (Tab one, Fig two)	HAMD21	24 ± 6 (10)	24 ± 6 (10)	22 ± 6 (10)	17 ± 9 (10)	2 ± 6 (10)	7 ± 8 (10)
								MADRS	30 ± 6 (10)	29 ± 6 (10)	29 ± 6 (10)	19 ± 9 (10)	1 ± 6 (10)	10 ± 8 (10)
Fitzgerald et al. (2003)	46	43%	+	+ 10%	N/A	+	All	MADRS	36 ± 8 (20)	37 ± 8 (40)	35 ± 8 (20)	32 ± 8 (40)	1 ± 8 (20)	5 ± 8 (40)
								BDI21	32 ± 9 (20)	34 ± 11 (40)	29 ± 9 (20)	27 ± 11 (40)	3 ± 9 (20)	7 ± 11 (40)
							Tab two 10Hz	MADRS	36 ± 8 (20)	36 ± 8 (20)	35 ± 8 (20)	31 ± 8 (20)	1 ± 8 (20)	5 ± 8 (20)
								BDI21	32 ± 9 (20)	33 ± 12 (20)	29 ± 9 (20)	27 ± 12 (20)	3 ± 9 (20)	6 ± 12 (20)
							Tab two 1Hz	MADRS	36 ± 8 (20)	38 ± 8 (20)	35 ± 8 (20)	32 ± 9 (20)	1 ± 8 (20)	6 ± 8 (20)
								BDI21	32 ± 9 (20)	35 ± 9 (20)	29 ± 9 (20)	27 ± 11 (20)	3 ± 9 (20)	8 ± 10 (20)
Höppner et al. (2003)	59	70%	N/A	+ 3%	–	+	Fig two 20Hz	HAMD21	25 ± 8 (10)	22 ± 4 (10)	13 ± 8 (10)	14 ± 5 (10)	12 ± 8 (10)	8 ± 5 (10)
							Fig one 20Hz	BDI21	28 ± 8 (10)	26 ± 7 (10)	22 ± 11 (10)	18 ± 8 (10)	6 ± 10 (10)	8 ± 8 (10)
Loo et al. (2003)	52	79%	some	+ 16%	N/A	+	Authors	HAMD17	20 ± 4 (10)	24 ± 5 (9)	16 ± 7 (10)	19 ± 8 (9)	4 ± 6 (10)	5 ± 7 (9)
								MADRS	33 ± 5 (10)	38 ± 6 (9)	27 ± 10 (10)	31 ± 14 (9)	6 ± 9 (10)	7 ± 12 (9)
Nahas et al. (2003)	43	61%	N/A	+ 100%	N/A	–	Authors	HAMD28	33 ± 8 (12)	32 ± 4 (11)	24 ± 12 (12)	24 ± 12 (11)	9 ± 11 (12)	8 ± 11 (11)
Buchholtz et al. (2004)	50	31%	N/A	+ 23%	N/A	+	Authors	HAMD17	24 ± 5 (7)	26 ± 3 (6)	13 ± 10 (7)	16 ± 3 (6)	11 ± 9 (7)	10 ± 3 (6)
Hausmann et al. (2004)	47	61%	N/A	N/A	–	+D1	Tab two	HAMD21	34 ± 4 (13)	32 ± 6 (25)	22 ± 8 (13)	18 ± 9 (25)	12 ± 7 (13)	14 ± 8 (25)
								BDI13	31 ± 12 (13)	32 ± 10 (25)	21 ± 14 (13)	17 ± 12 (25)	10 ± 13 (13)	15 ± 11 (25)
Holtzheimer et al. (2004)	43	47%	+	–	–	–	Tab two	HAMD17	21 ± 6 (8)	23 ± 5 (7)	15 ± 3 (8)	15 ± 3 (7)	6 ± 5 (8)	8 ± 4 (7)
								BDI21	28 ± 11 (8)	30 ± 10 (7)	22 ± 2 (8)	24 ± 3 (7)	6 ± 10 (8)	6 ± 9 (7)
Kauffmann et al. (2004)	52	92%	+	N/A	N/A	+	Text	HAMD21	18 ± 5 (5)	22 ± 6 (7)	12 ± 4 (5)	11 ± 8 (7)	6 ± 4 (5)	11 ± 7 (7)
Koerselman et al. (2004)	52	56%	N/A	N/A	N/A	+	Tab four	HAMD17	26 ± 6 (26)	26 ± 4 (26)	22 ± 7 (24)	21 ± 7 (25)	4 ± 7 (25)	5 ± 6 (26)
Mosimann et al. (2004)	62	42%	+	+ 17%	N/A	+	Tab three	HAMD21	24 ± 7 (9)	28 ± 5 (15)	20 ± 7 (9)	23 ± 7 (15)	4 ± 7 (9)	5 ± 6 (15)
								BDI21	28 ± 11 (9)	30 ± 9 (15)	23 ± 11 (9)	24 ± 13 (15)	5 ± 11 (9)	6 ± 12 (15)
Poulet et al. (2004)	43	47%	N/A	N/A	N/A	+D1	Authors	MADRS	36 ± 7 (9)	33 ± 5 (10)	17 ± 9 (9)	16 ± 8 (10)	19 ± 8 (9)	17 ± 7 (10)
								BDI13	18 ± 6 (9)	21 ± 8 (10)	11 ± 7 (9)	14 ± 7 (10)	7 ± 7 (9)	7 ± 8 (10)
Rossini et al. (2005)^a^	47	80%	–	–	–	+D1	Tab two	HAMD21					8 ± 7 (47)	13 ± 7 (49)
Rumi et al. (2005)	39	85%	N/A	N/A	–	+	Fig two	MADRS	39 ± 8 (24)	38 ± 8 (22)	28 ± 12 (24)	14 ± 11 (22)	11 ± 11 (24)	24 ± 10 (22)
Su et al. (2005)	43	73%	+	+ 17%	–	+	All (Tab two)	HAMD21	23 ± 5 (10)	24 ± 7 (20)	19 ± 8 (10)	11 ± 7 (20)	4 ± 7 (10)	13 ± 7 (20)
								BDI21	33 ± 10 (10)	31 ± 9 (20)	29 ± 15 (10)	16 ± 10 (20)	4 ± 13 (10)	15 ± 10 (20)
Avery et al. (2006)	44	54%	+	–	–	+	Tab one, Text	HAMD17	24 ± 3 (33)	24 ± 4 (35)	20 ± 6 (33)	16 ± 8 (35)	4 ± 5 (33)	8 ± 7 (35)
								BDI21	28 ± 8 (33)	28 ± 9 (35)	24 ± 8 (33)	17 ± 13 (35)	4 ± 8 (33)	11 ± 12 (35)
Fitzgerald et al. (2006)	45	62%	+	+ 16%	–	+	Authors, Tab two	HAMD17	20 ± 5 (22)	23 ± 7 (25)	17 ± 6 (22)	16 ± 7 (25)	3 ± 6 (22)	7 ± 7 (25)
								BDI21	29 ± 10 (22)	29 ± 10 (25)	22 ± 14 (22)	18 ± 10 (25)	7 ± 12 (22)	11 ± 10 (25)
								MADRS	34 ± 6 (22)	34 ± 6 (25)	31 ± 8 (22)	26 ± 10 (25)	3 ± 7 (22)	8 ± 9 (25)
Garcia-Toro et al. (2006)	48	55%	+	–	–	+	Tab two	HAMD21	25 ± 7 (10)	27 ± 5 (10)	24 ± 8 (10)	20 ± 8 (10)	1 ± 8 (10)	7 ± 7 (10)
Januel et al. (2006)	38	78%	–	–	N/A	–	Tab one	HAMD17	22 ± 3 (16)	22 ± 4 (11)	17 ± 5 (16)	10 ± 6 (11)	5 ± 4 (16)	12 ± 5 (11)
Anderson et al. (2007)	47	55%	some	N/A	N/A	+	Tab one	MADRS	28 ± 7 (14)	27 ± 4 (11)	23 ± 10 (14)	15 ± 10 (11)	5 ± 9 (14)	12 ± 9 (11)
Bortolomasi et al. (2007)	56	58%	N/A	+ 16%	N/A	+	Fig two	HAMD24	22 ± 4 (7)	25 ± 8 (12)	18 ± 5 (7)	11 ± 10 (12)	4 ± 5 (7)	14 ± 9 (12)
							Fig one	BDI21	27 ± 5 (7)	26 ± 7 (12)	22 ± 6 (7)	12 ± 10 (12)	5 ± 6 (7)	14 ± 9 (12)
Herwig et al. (2007)	50	60%	some	+ 6%	N/A	+D1	Tab one	HAMD21	23 ± 5 (65)	25 ± 5 (62)	14 ± 8 (65)	14 ± 6 (62)	9 ± 7 (59)	11 ± 6 (57)
							Tab two							
								BDI21	27 ± 10 (65)	27 ± 9 (62)	18 ± 10 (65)	16 ± 9 (62)	9 ± 10 (59)	11 ± 9 (57)
								MADRS	27 ± 6 (65)	28 ± 7 (62)	16 ± 9 (65)	17 ± 8 (62)	11 ± 8 (59)	11 ± 8 (57)
Loo et al. (2007)	48	47%	–	+ 11%	–	+	Tab two	HAMD17	21 ± 4 (19)	19 ± 4 (19)	15 ± 7 (19)	12 ± 6 (19)	6 ± 6 (19)	7 ± 5 (19)
								BDI21	34 ± 8 (19)	27 ± 8 (19)	27 ± 10 (19)	18 ± 10 (19)	7 ± 9 (19)	9 ± 9 (19)
								MADRS	33 ± 4 (19)	30 ± 4 (19)	27 ± 10 (19)	19 ± 8 (19)	6 ± 9 (19)	11 ± 7 (19)
O’Reardon et al. (2007)	48	53%	some	–	–	–	Tab one	HAMD17	23 ± 4 (146)	23 ± 3 (155)	19 ± 6 (146)	17 ± 6 (155)	4 ± 5 (146)	6 ± 5 (155)
								MADRS	34 ± 6 (146)	33 ± 6 (155)	30 ± 10 (146)	27 ± 11 (155)	4 ± 9 (146)	6 ± 10 (155)
Stern et al. (2007)	53	–	some	–	–	–	All (Tab three)	HAMD21	27 ± 3 (15)	28 ± 4 (30)	27 ± 4 (14)	19 ± 8 (28)	0 ± 4 (14)	9 ± 7 (29)
							10Hz		27 ± 3 (15)	28 ± 3 (10)	27 ± 4 (14)	15 ± 6 (10)	0 ± 4 (14)	13 ± 5 (10)
							1Hz L		27 ± 3 (15)	28 ± 4 (10)	27 ± 4 (14)	28 ± 6 (8)	0 ± 4 (14)	0 ± 5 (9)
							1Hz R		27 ± 3 (15)	28 ± 4 (10)	27 ± 4 (14)	16 ± 5 (10)	0 ± 4 (14)	12 ± 5 (10)
Bretlau et al. (2008)	55	62%	some	N/A	–	+D1	Tab two	HAMD17	25 ± 3 (23)	25 ± 3 (22)	19 ± 5 (23)	16 ± 4 (22)	6 ± 4 (23)	9 ± 4 (22)
Mogg et al. (2008)	54	63%	some	+ 2%	N/A	+	Authors	HAMD17	22 ± 5 (30)	21 ± 5 (29)	20 ± 8 (29)	16 ± 9 (28)	2 ± 7 (30)	5 ± 8 (28)
								BDI-II21	36 ± 10 (30)	38 ± 11 (29)	31 ± 15 (26)	26 ± 15 (28)	5 ± 13 (28)	12 ± 14 (28)

Notes: All studies included patients with a major depressive episode and/or disorder according to DSM-IV and/or ICD-10 criteria. The mean number of patients per group was used in the final calculations if patients dropped out throughout the study between baseline and final sessions. All values ending with exactly 0.5 were rounded as follows to reduce the rounding error: zero and uneven numbers upwards (1.5 = 2), even numbers downwards (2.5 = 2). Standard error of the mean (*SEM*) was converted to standard deviation (*SD*) using the formula: *SD = SEM × √N* (where *N* = sample size of sham or rTMS groups). ‘All’ indicates that scores for all independent subgroups within studies were combined. ^A^Treatment-resistance: + are studies in which all patients failed (or showed intolerance to) ≥2 antidepressant trials (of same or different class) of an adequate dose/length during current or lifetime episode; − are studies in which all patients failed ≤1 antidepressant trials; ‘some’ are studies in which patients failed ≥1 antidepressant trial (these studies were excluded from all analyses because this category overlapped with the + and – categories); ^B^Bipolar (%): + are studies including any proportion of patients with bipolar disorder at baseline; − means that all patients had unipolar depression (no history of bipolar disorder, mania, hypomania, Axis I disorders); ^C^Psychotic (%): + are studies including any proportion of patients with psychotic features at baseline; − means that all patients had non-psychotic depression (no history of psychosis, Axis I disorders); ^D^Medication = antidepressants (+means any proportion of patients/study received stable doses, +D1 means that antidepressants were started on day 1 concurrently with rTMS, − means that all patients were unmedicated but some might have received mood stabilizers); ^E^It was assumed that HAMD21 or BDI21 were used if no further information was provided.; ^F^’Last session’ refers to the last session of the double-blind phase of a study. ^a^Depression scores were reported as change scores from baseline (baseline – final session). Abbreviations: BDI, Beck Depression Inventory; D1, antidepressants started on day 1 concurrently with rTMS; DLPFC, dorsolateral prefrontal cortex; DSM-IV, Diagnostic and Statistical Manual of Mental Disorders; Fig, Figure; HAMD, Hamilton Depression Rating Scale; ICD-10, International Statistical Classification of Diseases and Related Health Problems; L, left DLPFC; MADRS, Montgomery Åsberg Depression Rating Scale; N/A, not reported or inadequate information; R, right DLPFC; RCT, randomised-controlled trial; rTMS, repetitive transcranial magnetic stimulation; *SD*, standard deviation; Tab, Table.

### Meta-Analysis

The mathematical approach used in the current meta-analysis is explained in detail in the Additional file 1. In general, the current study utilised the random-effects model of meta-analysis with inverse-variance weights (Borenstein et al. 2009) using Comprehensive Meta-Analysis 2.0 (CMA; Biostat Inc., USA) and SPSS-21 (IBM Corp., USA). The random-effects model was chosen because it was assumed that

1. the primary studies included in the current analysis were a random sample of all studies on the topic,
2. the effect sizes of those studies would differ based on the heterogeneous rTMS parameters and/or clinical characteristics of patients (Tables 1 and 2),
3. results from studies in the current meta-analysis could be extrapolated to a wider population of patients with major depression.

One important assumption of any meta-analysis is that each study is independent of all other studies in the analysis and thus contributes only one effect size to the computation of the overall mean weighted effect size (Borenstein et al. 2009). Therefore, if studies used multiple rTMS groups with different parameters (such as two high frequencies of 5 Hz and 20 Hz), then the depression scores from both rTMS groups were combined into one (for formulae see the Additional file 1).

In the first step of the analysis, one effect size was computed for each study. The effect size used in the current meta-analysis was the standardised mean difference (Cohen’s *d*), which was computed as follows:

$$ d = sham\ \left( mean\  standardised\  depression\  score\  at\  baseline\ \hbox{--}\ final\  session\right)\ \hbox{--}\ active\  rTMS\ \left( mean\  standardised\  depression\  score\  at\  baseline\ \hbox{--}\ final\  session\right). $$

The interpretation criteria for the absolute size of Cohen’s *d* are: *d* = .20-.49 (low), *d* = .50-.79 (moderate), and *d* ≥ .80 (high) (Cohen 1988). Since Cohen’s *d* is often inflated in studies conducted on small samples, a standardised mean difference corrected for the sample size, Hedges’ *g*, was also computed (Borenstein et al. 2009); for the formula refer to the Additional file 1.

In the second step of the analysis, each effect size was weighted based on the inverse of the sum of the within- and between-study variance (DerSimonian and Laird 1986). The logic behind this weighing method is that studies with a high variability of scores (high variance, low precision) contribute only a small weight to the overall mean weighted effect size and vice-versa.

In the final step of the analysis, one overall mean weighted effect size of all studies was computed as the sum of the product of all effect sizes and weights divided by the sum of all weights (Borenstein et al. 2009). According to our calculation, *negative* values of the overall mean weighted effect sizes (*d* or *g* and their 95% confidence intervals, *95% CIs*) indicate that depression scores are reduced on the final session compared to baseline, favouring rTMS over sham.

Heterogeneity among effect sizes was tested using a *Q* statistic and an *I*^*2*^ index (Borenstein et al. 2009). The *Q* statistic tests the null-hypothesis that there is homogeneity among effect sizes in the analysis (*Q* = 0). However, the interpretation of the null-hypothesis testing is prone to Type I and Type II statistical errors and thus cannot be used as a reliable measure of heterogeneity alone. Instead, the *Q* statistic can be expressed on a 0-100% scale using the so-called *I*^*2*^ index (*I*^*2*^ = 100% × (*Q*-*df*)/*Q* with *df* = *N*-1; *N* = number of studies). The *I*^*2*^ index can be interpreted as the variability in effect sizes due to real differences among studies (as opposed to chance) using the following criteria: 25% (low heterogeneity), 50% (moderate heterogeneity), and 75% (high heterogeneity) (Higgins et al. 2003).

### Sensitivity and moderator analyses

The stability of the overall mean weighted effect size over time was investigated as one study at a time was added to all previous studies (cumulative analysis) and as one study at a time was removed from the overall analysis (one-study removed analysis). The moderator analyses were used to compare the mean weighted effect sizes between subgroups of studies with similar characteristics (univariate subgroup analyses) and to predict change in the weighted effect sizes based on continuous characteristics of studies (univariate meta-regressions).

### Publication bias analyses

Publication bias occurs when the overall mean weighted effect size is inflated in a meta-analysis due to a selection of studies biased towards those with larger (and statistically significant) effect sizes (Borenstein et al. 2009). Although a novel literature search was not conducted in the current study, publication bias was assessed using methods available in the CMA software. The Rosenthal’s Fail-Safe *N* (Rosenthal 1979) was used to compute the theoretical number of unpublished studies with low effect sizes required to remove the significance of the overall mean weighted effect size. The Duval and Tweedie’s Trim-and-Fill analysis (Duval and Tweedie 2000) was used to test if effect sizes plotted against their variability (standard error of the mean, *SEM)* on a so-called funnel plot (Sterne and Egger 2001) are symmetrically distributed around the overall mean weighted effect size. Finally, the Begg and Mazumdar Rank Order Correlation (Kendall’s *tau b*) between the standardised effect sizes vs. *SEM* in each study (Begg and Mazumdar 1994) and the Egger’s regression of 1/*SEM* (predictor) on the standardised effect sizes (Egger et al. 1997) were used to test if studies with lower effect sizes differ systematically (significantly) from studies with higher effect sizes. It was assumed that publication bias is present if Fail-Safe *N* is low, the funnel plot is asymmetrical, Begg and Mazumdar correlation is significant, and the intercept of Egger’s regression line significantly deviates from zero (Borenstein et al. 2009).

## Results

The *N =* 40 primary RCTs included in the current meta-analysis were conducted in 15 countries, mostly in Western Europe (*N* = 20 RCTs, 50%), USA (*N* = 13 RCTs, 32%), and Australia (*N* = 6 RCTs, 15%). According to the overall analysis, there was a significant reduction in the mean depression scores from baseline to final, favouring rTMS over sham, in *N* = 40 RCTs based on a total of 1583 patients (844 in the active rTMS and 739 in sham groups; for the forest plot see Additional file 1: Figure S1). However, the magnitude of such an overall short-term antidepressant effect of rTMS was only moderate (the overall mean weighted effect size *d* = −.54, *95% CI*: −.68, −.41; *p*_*two-tailed*_ < .001 and *g* = −.53; *95% CI*: −.66, −.40; *p*_*two-tailed*_ < .001). Since *d* and *g* were similar in magnitude, it is unlikely that *d* was inflated in the mostly small-sample primary studies included in this analysis. Thus, all subsequent analyses were performed using Cohen’s *d* alone.

There was little heterogeneity among the 40 effect sizes due to real (methodological) differences among studies (*Q =* 54*, df =* 39*, p*_*two-tailed*_ = .054, *I*^*2*^ = 28%). The overall effect size was low-moderate as studies were added over time cumulatively (Additional file 1: Figure S2) and was not dependent on any one study alone (as one study at a time was removed from the analysis; Additional file 1: Figure S3). It is also unlikely that publication bias occurred because Fail-Safe *N* of 908 was high and Begg and Mazumdar correlation and Egger’s regression were not statistically significant (*p*_*two-tailed*_ = .633 and *p*_*two-tailed*_ = .112 respectively). Although the funnel plot was not symmetrical (Additional file 1: Figure S4), the overall mean weighted *d* corrected for seven studies theoretically missing from the analysis indicated that antidepressant effect was still present in the data favouring rTMS over sham (corrected overall mean weighted *d* = −.42, *95% CI*: −.57, −.28).

The short-term antidepressant effect favouring rTMS over sham was observed when studies were grouped according to each depression scale separately: HAMD used in 36 (90%) RCTs (the overall mean weighted *d* = −.54, *95% CI*: −.69, −.40; *p*_*two-tailed*_ < .001), BDI used in 17 (42%) RCTs (the overall mean weighted *d* = −.42, *95% CI*: −.58, −.26; *p*_*two-tailed*_ < .001), and MADRS in 12 (30%) RCTs (the overall mean weighted *d* = −.44, *95%CI*: −.69, −.20; *p*_*two-tailed*_ < .001).

The *N* = 40 RCTs utilised the following combinations of frequency-location of rTMS: HFL in *N* = 33 (82%) RCTs, LFR in *N* = 5 (12%) RCTs, bilateral or sequential (left then right) in *N* = 4 (10%) RCTs, and low-frequency left in *N* = 3 (8%) RCTs. Inspection of the 33 effect sizes in HFL studies revealed that one RCT (Stern et al. 2007) produced a significantly higher effect size (*d* = −2.93) compared to all other 32 RCTs (*d* = −.47) and thus was classified as a statistical outlier. Since the inclusion of this study would inflate all effect sizes in the HFL analysis, this study was removed from all subsequent analyses to maintain statistical conservativeness (for more details see Additional file 1: Figure S5; note that the overall effect size based on all three active rTMS subgroups in this RCT was not classified as an outlier and thus the study was kept in the overall analysis of *N* = 40 RCTs presented above). The short-term antidepressant effect favouring rTMS over sham was observed in HFL studies (the overall mean weighted *d* = −.47, *95% CI*: −.61, −.33; *p*_*two-tailed*_ < .001; *N* = 32 RCTs), LFR studies (the overall mean weighted *d* = −1.21, *95% CI*: −1.85, −.56; *p*_*two-tailed*_ < .001; *N* = 5 RCTs), and bilateral or sequential studies (the overall mean weighted *d* = −.45, *95% CI*: −.82, −.09; *p*_*two-tailed*_ = .015; *N* = 4 RCTs) but not in the low-frequency left rTMS studies (the overall mean weighted *d* = −.35, *95% CI*: −.97, .27; *p*_*two-tailed*_ = .268; *N* = 3 RCTs). Due to a low number of studies in the other subgroups, further analyses were conducted only on the largest subgroup of HFL studies (*N* = 32 RCTs).

**Figure 2:**
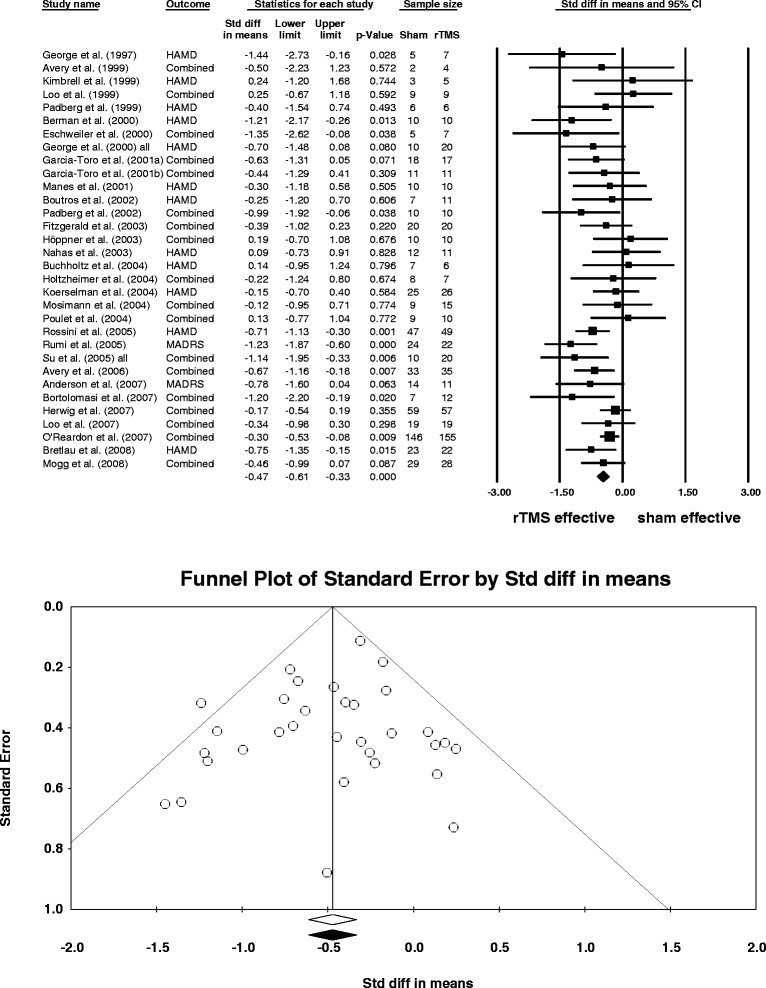
**Random-effects meta-analysis of depression scores (baseline-final) after HFL rTMS compared to sham in** ***N*** **= 32 studies.** Notes: ‘All’ refers to all patients in two HFL rTMS groups who received rTMS with two different stimulation frequencies. ‘Combined’ in the column ‘Outcome’ indicates that more than one depression scale was used in a study and the effect sizes according to the multiple scales were combined into one. The mean number of patients per group was used in the final calculations if patients dropped out throughout the study between baseline and final sessions. The forest plot (top) shows the weighted effect size *d* (box) and its 95% *CI* (vertical line through the box) for each study in the analysis. The diamond depicts the overall mean weighted *d* of all studies and its 95% *CI* (width of the diamond). The mean depression scores (baseline – final) were significantly reduced after HFL rTMS compared to sham in 32 studies (overall mean weighted *d* = −.47, 95% *CI*: −.61, −.33). The funnel plot (bottom) shows the effect size *d* versus standard error in each study in the analysis. The plot was symmetrical around the overall mean weighted *d* suggesting that there was little evidence for publication bias in the current meta-analysis. Abbreviations: *CI*, confidence interval; HAMD, Hamilton Depression Rating Scale; HFL, high-frequency left rTMS; MADRS, Montgomery Åsberg Depression Rating Scale; rTMS, repetitive transcranial magnetic stimulation; Std diff, standardised mean difference (Cohen’s *d*).

The antidepressant effect favouring HFL rTMS over sham in 32 RCTs was based on 1279 patients (Figure 2). There was little heterogeneity among the 32 effect sizes attributable to real differences among HFL studies (*Q =* 39*, df =* 31*, p*_*two-tailed*_ = .154, *I*^*2*^ = 20%). The overall effect size was consistently low-moderate as studies were added over time and was not dependent on any one study alone (for cumulative and one-study removed analyses see the Additional file 1: Figures S6 and S7). It is unlikely that publication bias occurred because Fail-Safe *N* of 425 was high, funnel plot was symmetrical (Figure 2), and Begg and Mazumdar correlation and Egger’s regression were not statistically significant (*p*_*two-tailed*_ = .808 and *p*_*two-tailed*_ = .322 respectively).

Grouping of HFL studies based on the clinical characteristics of patients revealed that the majority of those studies included patients with treatment-resistance, on antidepressants (at stable doses in *N* = 20 RCTs or started concurrently with rTMS in *N* = 5 RCTs), with bipolar depression, and without psychotic features (Table 3). The proportions of bipolar and psychotic patients per study were mostly low (<50%) except for one study conducted on bipolar patients only (Table 2). Most patients in the HFL studies were middle-aged or older (39–62 years old on average) and about half were female (Table 3). The most common rTMS parameters in the HFL studies were: frequency of 10 Hz, stimulus intensity equivalent to the resting motor threshold of 80-110%, 10 stimulation sessions, 1600 stimuli/session (or 16000 stimuli/study), 20 trains/session, 30 s inter-train interval, and a 70 mm coil diameter (Table 3). Most studies used the figure-of-eight shape of the stimulating coil and a 90 degree angle from scalp during sham (Table 3).

**Table 3:** **Univariate random-effects subgroup analyses and meta-regressions in** ***N*** **= 32 HFL rTMS studies**

**Study subgroups** ^**a**^	***N*** **studies (%)** ^**b**^	***d (95% CI)***	***p***_***two-tailed***_	**Meta-regression predictors**	***N*** **studies** ^**b**^	**Mode**	**Range**	**Regression line slope** ***p***_***two-tailed***_
Treatment resistance	13			Patient characteristics				
YES (all failed ≥2 AD trials)	10 (77%)	-.56 (−.81, −.32)	<.001*	Female patients (%)	32	47, 50	22-92	.003*
NO (all failed ≤1 AD trials)	3 (23%)	-.58 (−.90, −.26)	<.001*	Mean age (years)	32	43, 44	39-62	.194
Antidepressants	32			Bipolar patients (%)	24	0	0-100	.211
YES (any % of patients)	25 (78%)	-.51 (−.68, −.35)	<.001*	Psychotic patients (%)	14	0	0-8	.789
Stable dose	20 (80%)	-.54 (−.74, −.34)	<.001*	rTMS parameters				
Started with rTMS	5 (20%)	-.43 (−.75, −.12)	.007*	Frequency (Hz)	32	10	5-20	.824
NO (all patients)	7 (22%)	-.33 (−.53, −.14)	.001*	Motor threshold (%)	32	80, 90, 110	80-120	.984
Bipolar depression	24			Total sessions	32	10	5-20	.813
YES (any % of patients)	16 (67%)	-.39 (−.62, −.16)	.001*	Stimuli/session	20	1600	250-3000	.021*^c^
NO (all patients)	8 (33%)	-.45 (−.62, −.28)	<.001*	Total stimuli/study	20	16000	1250-60000	.124
Psychotic depression	14			Trains/session	28	20	5-100	.217
YES (any % of patients)	3 (21%)	-.73 (−1.78, .33)	.177	Inter-train interval (s)	31	30	8-60	.680
NO (all patients)	11 (79%)	-.59 (−.81, −.36)	<.001*					
Coil-type	30							
Figure-of-eight	28 (93%)	-.50 (−.66, −.34)	<.001*					
Circular	2 (7%)	-.59 (−1.59, .42)	.254					
Coil angle sham	32							
0° (inactive coil)	2 (6%)	-.33 (−.85, .19)	.217					
0° (sham coil)	5 (16%)	-.63 (−.98, −.28)	<.001*					
45°	12 (38%)	-.27 (−.50, −.04)	.022*					
90°	13 (41%)	-.61 (−.82, −.40)	<.001*					

Notes: ^a^There were no statistically significant differences in effect sizes between subgroups. Subgroups were compared using the mixed-effect model (random-effects model was used to compute the overall mean weighted *d* in each subgroup, overall mean weighted *d* of subgroups were compared using the fixed-effect model because the number of subgroups was fixed). ^b^Not all studies reported the characteristics investigated in this table. ^c^Unlike % female patients, the slope of regression line of stimuli/session on weighted *d* was driven by the largest study in the current analysis (O’Reardon et al. 2007). After removal of this study the *p*-value of the slope of regression line was .061. Abbreviations: AD, antidepressant; *d*, weighted standardised mean difference (Cohen’s *d*); HFL, high-frequency left rTMS; rTMS, repetitive transcranial magnetic stimulation. *p < .05

According to our univariate classification of studies, the antidepressant effect favouring HFL rTMS over sham was independent of treatment-resistance, treatment with antidepressants, and bipolar diagnoses (Table 3). The antidepressant effect was also present in studies with non-psychotic patients and in studies utilising figure-of-eight coils (Table 3). The magnitude of the antidepressant effect was similar in studies with sham coils and coils tilted at 90 degrees from scalp (Table 3).

**Figure 3:**
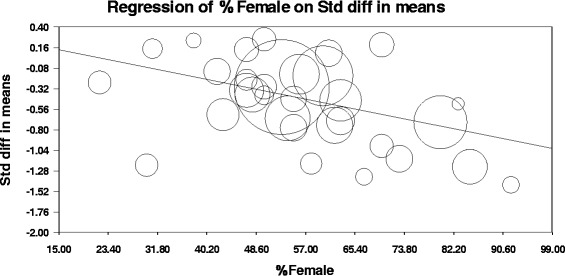
**Univariate random-effects meta-regression of % female patients (predictor) on the weighted effect sizes** ***d*** **(outcome) in** ***N*** **= 32 HFL rTMS studies.** Notes: The figure shows a scatterplot of weighted *d*/study (Y-axis) versus proportion of female patients/study (X-axis). The circles depict individual studies (the larger the diameter of the circle the larger the study weight). The slope of the regression line (*B* = −.01) was statistically significant (*p*_*two-tailed*_ = .003) indicating that depression scores were significantly reduced after HFL rTMS compared to sham in studies with higher proportion of female patients. The predictor (% female patients) explained 97% of the between-study variance in weighted *d* according to the following formula: *R* ^*2*^ = 1-(*T* ^*2*^ *model/T* ^*2*^ *total*), where *T* ^*2*^ *model* (here = .00093) is the between-study variance in the weighted *d* unexplained by the regression model containing the predictor and *T* ^*2*^ *total* (here = .029) is the within- and between-study variance. The removal of the study with the largest weight (O’Reardon et al. 2007) did not change the outcome of this analysis (*B* = −.01, slope *p*_*two-tailed*_ = .006; for a scatterplot see the Additional file 1). Abbreviations: HFL, high-frequency left rTMS; rTMS, repetitive transcranial magnetic stimulation; Std diff, standardised mean difference (Cohen’s *d*).

Except for one, all univariate random-effects meta-regressions were not statistically significant. Thus, the mean weighted *d* per study could not be univariately predicted by any of the following study characteristics in HFL rTMS studies: mean age of all patients per study, frequency of stimulation, stimulus intensity (% motor threshold), number of sessions, stimuli/session, stimuli/study, trains/session, and inter-train interval. However, a significantly higher antidepressant effect was observed in HFL studies with higher proportion of female patients (Table 3, Figure 3). The predictor (% female patients) explained 97% of the between-study variance in effect sizes in the HFL rTMS studies (Figure 3).

## Discussion

The results of the current meta-analysis quantitatively supplement the narrative findings of Dell’Osso and colleagues (2011). Specifically, we confirm that rTMS had a significant, but only moderate, short-term antidepressant effect in the treatment of major depression based on data from *N* = 40 RCTs published between 1997–2008 and selected from 13 past meta-analyses on this topic (published until 2010). This result is based on data from 1583 patients tested in 15 countries. A similar result was also observed based on the newer RCTs published between 2010–2013 (Kedzior et al. 2014). Although the clinical relevance of a moderate effect size is questionable, the antidepressant effect of rTMS was relatively robust as new studies were added to the existing ones (from 2000–2008). It remains to be seen if rTMS also has stable longitudinal antidepressant properties. The moderate effect size is probably unrelated to heterogeneous statistical approaches used in the past meta-analyses because it was also obtained in the current study conducted using one method of meta-analysis.

The current study shows that depression scores were reduced after rTMS regardless of depression scale used. Thus, the often self-administered BDI scale appears to be as effective at measuring depression as the widely used HAMD that is clinician-administered and has the best psychometric properties of the three scales (Trajkovic et al. 2011). However, only moderate effect sizes might have resulted from combining depression scores from all scales and/or different versions of HAMD and BDI scales (Table 2) in the current analysis. Thus, if adequate number of studies is available, future meta-analyses should be conducted on data based on one version of the same scale to reduce the variability of depression scores and possibly increase the overall effect sizes.

Similarly to the other meta-analyses (Dell’Osso et al. 2011), the current results indicate that the short-term antidepressant effect was observed in studies utilising HFL rTMS and also LFR and bilateral designs (Table 3). The LFR and bilateral stimulation need to be investigated in more primary studies to reach consensus about their clinical effectiveness similar to that of HFL designs (Fitzgerald and Daskalakis 2012). In general, while a single study design might not be used as paradigmatic (Herrmann and Ebmeier 2006), combinations of various rTMS parameters appear to facilitate the antidepressant properties of rTMS.

The novel, unexpected finding of the current meta-analysis is that the antidepressant effect of HFL rTMS was higher in RCTs with higher proportion of female patients (Figure 3). To our knowledge this effect was not tested in any of the past 13 meta-analyses nor in the newer meta-analyses published since, except for our follow-up analysis to the current study (for review see Kedzior et al. 2014). According to our follow-up meta-analysis, the short-term antidepressant effect of rTMS with any frequency/location was also higher with higher proportion of female patients in *N* = 53 RCTs published between 1997–2013 (Kedzior et al. 2014). The finding that the response to rTMS might depend on gender is particularly interesting because twice as many women as men are diagnosed with depression worldwide (Bromet et al. 2011). However, this result needs to be interpreted with some caution because the univariate meta-regression analysis did not control for any other possible confounders. Therefore, it is possible that female patients systematically differed from the male patients in terms of age, severity of resistance, or design/rTMS parameters of the study in which they have participated. Thus, the apparent relationship between gender and effect size could be secondary to those and other factors not taken into account in our analysis. In support of this argument, the open-label studies and smaller RCTs in medicated or unmedicated patients showed that young age and lower severity of treatment resistance were associated with an improved antidepressant outcome of rTMS controlling for other predictors (Brakemeier et al. 2007; Brakemeier et al. 2008; Fregni et al. 2006) and responders to rTMS were significantly younger than non-responders (Kozel et al. 2000). In female patients reduction in depression was associated with young age and a stage of menstrual cycle (Huang et al. 2008). Treatment-resistant, unipolar female patients also showed both a short-term and also a long-term (up to 24 weeks) antidepressant response to the combination of 10 sessions of LFR rTMS and two sessions of partial sleep deprivation (Krstic et al. 2014). A better outcome with lower degree of treatment resistance in the current episode was observed univariately in non-psychotic unipolar patients according to a large RCT (O’Reardon et al. 2007) and an open-label extension trial (Lisanby et al. 2009). The open-label extension of another large RCT (George et al. 2010) showed that extending the stimulation period for more than five weeks and changing the stimulation site improved remission rates in patients who failed to meet the minimal response criteria during the RCT (McDonald et al. 2011). One way of dealing with possible multiple predictors of the antidepressant response to rTMS would be to include such predictors in a multivariate meta-regression analysis. However, such an analysis was not conducted here because not all studies reported all their patient/rTMS characteristics (see Tables 1–3) leading to missing values on many predictors and thus a low ratio of studies to possible predictors. In fact, the effect sizes should be compared between male and female patients while controlling for multiple predictors in the future RCTs due to availability of individual patient data rather than group data used in meta-analyses.

It is unlikely that the significant meta-regression was due to a statistical artifact and/or the largest RCT (O’Reardon et al. 2007) in the current meta-analysis. Compared to a traditional (unweighted) regression analysis, the slope of regression line was influenced by study weights rather than effect sizes alone in the current analysis. Therefore, studies with higher precision (and weight) had a higher influence on the slope of the regression line than (presumably) lower-quality studies with a high variability of scores. The plot of the weighted effect sizes versus the proportion of female patients (Figure 3) suggests that the relationship between the two variables was reasonably linear and not influenced by any major outliers. The outcome of the analysis also remained unchanged after the study with the largest weight (O’Reardon et al. 2007) was removed from the analysis (see the Additional file 1: Figure S8). Finally, the predictor ‘proportion of female patients/study’ explained a high amount (97%) of between-study variance in the current analysis.

The relatively low effect sizes in the current and other meta-analyses could have been due to differences in the sham conditions and thus different levels of blinding integrity. Such integrity is important because the effect sizes in our analysis were computed based on the change in depression scores from baseline to final in rTMS compared to sham conditions. A recent meta-analysis of blinding integrity showed that only very few studies reported their blinding success and that the development of novel sham strategies, such as shielded magnetic coils, might help to adequately conceal the treatment allocation (Berlim et al. 2013). Based on our data, the most commonly used sham technique was tilting the active coil at the 90 degree angle from scalp in studies published between 1997–2008. Although it has been argued that tilted active coils could cause some cortical stimulation (Mitchell and Loo 2006), the current results showed that the effect sizes were similar in studies with sham coils (overall *d* = −.63) and coils tilted at 90 degree angle from scalp (overall *d* = −.61; Table 3) not controlling for other study characteristics. Thus, it remains to be seen if better blinding integrity could contribute to higher antidepressant effect of rTMS in the future RCTs.

The antidepressant effect of HFL rTMS was probably not secondary to concurrent treatment with antidepressants because it was observed in a group of studies that included unmedicated patients and in studies with treatment resistant patients. In the latter studies, rTMS might have acted as a ‘key-like’ mechanism by unlocking the unresponsive pathways in the DLPFC and beyond, and thus aiding the action of antidepressants. In general, the high-frequency rTMS is thought to reverse the hypo-excitability of the left DLPFC (Daskalakis et al. 2008). However, since HFL rTMS improves cognitive functioning in patients with depression, rTMS might also affect deeper neural areas beyond the stimulation site involved in aetiology of depression (Kedzior et al. 2012). Rather than affecting any particular structures, rTMS could aid the action of antidepressants by altering the circuit level connectivity because depression is not associated with abnormalities at any specific location in the brain (Pandya et al. 2012). For example, depending on the number of sessions, stimuli, and frequency, rTMS could induce changes in cortical inhibition or excitation by modifying synaptic release or reuptake of neurotransmitters targeted by antidepressants depending on their class (de Jesus et al. 2014; Medina and Tunez 2013). The response to antidepressants in rTMS studies might also depend on the waveform of stimuli (biphasic vs. monophasic) that are associated with differential changes in cortical excitability (Groppa et al. 2012; Loo and Mitchell 2005).

The reduction in depression after HFL rTMS might have been concurrent or even secondary to improvements in cognitive functioning (Kedzior et al. 2012). Indeed, a systematic review showed that rTMS characteristics, such as 10–20 Hz frequency, 10–15 sessions, and stimulus intensity with 80-110% resting motor threshold were associated with cognitive improvements in psychiatric disorders (Guse et al. 2010). These characteristics were also commonly used in the HFL rTMS studies in the current meta-analysis. Importantly, the cognitive effects of rTMS depended on the correct positioning of the coil (Guse et al. 2010), a factor that was not controlled for in the current meta-analysis. According to Table 1, most studies included in the current analysis used the ‘5 cm’ rule for coil positioning. Using other localisation methods, such as the magnetic resonance image (MRI)-guided neuro-navigation, could further improve the antidepressant effect of rTMS (Rusjan et al. 2010).

There were a number of limitations in the current meta-analysis. Firstly, non-response rates and/or drop-out rates were not considered in our analyses. However, if reported, depression scores based on the intention-to-treat analysis were used in the current study. Secondly, treatment resistance should be defined as a ‘failure to respond to at least two antidepressants of *different classes* during *current* episode of depression’ (Berlim and Turecki 2007). Due to inadequate information provided in some studies we have used a more liberal version of this definition to classify treatment resistance (failure to respond to at least two antidepressants of the *same or different classes* during *any current or past* episode of depression). Thirdly, we did not control for other medications and/or affective disorders although evidence from a large RCT suggests that absence of comorbid anxiety is associated with an improved outcome of rTMS (Lisanby et al. 2009). Finally, the current analysis did not formally assess all measures of quality of studies according to The Cochrane Collaboration (Higgins et al. 2011). These measures are seven evidence-based domains (random sequence generation, allocation concealment, blinding of participants and personnel, blinding of outcome assessment, incomplete outcome data, selective reporting, and other bias) that should be assessed using a three point system (low risk, high risk, unclear risk) to evaluate the risk of bias (or quality of studies) included in systematic reviews. Three of these seven domains (randomisation, blinding, and withdrawals/drop-outs) can also be quantitatively assessed using the Jadad Scale, which has acceptable psychometric properties (construct validity and inter-rater agreement) (Jadad et al. 1996). A review of 965 systematic reviews (published between 1995–2002) revealed that no consensus exists with regards to the assessment of quality of primary studies (Moja et al. 2005). Although 94% of 809 Cochrane systematic reviews indeed assessed the quality of studies (compared to only 60% of non-Cochrane reviews), only approximately 50% of all reviews linked such an assessment to the results of their analyses (Moja et al. 2005). The current study utilised all formal approaches to the assessment of quality of studies as those most commonly applied in the 965 systematic reviews: explicitly listed exclusion/inclusion criteria, exploration of heterogeneity (subgroup analyses, meta-regressions), sensitivity analysis (identification of statistical outliers, one-study removed, and cumulative analyses), and a weighing method favouring studies with higher precision (Moja et al. 2005). The primary studies included in our analysis were of high quality based on the specific inclusion criteria: randomisation (including inactive sham group), blinding (sham stimulation applied at the same location as active rTMS), and reduction in other biases (carry-over effects eliminated due to inclusion of parallel stimulation data). We have addressed the difficulties with double-blinding of patients and staff in rTMS research by comparing the results based on different methods of blinding in the subgroup analysis (Table 3). Finally, the quality of studies was measured indirectly using the inverse-variance weighing method: Studies with higher variability of depression scores (and presumably lower quality) had lower weights and thus a low contribution to the magnitude of the overall antidepressant effect of rTMS and vice-versa.

## Conclusions

Daily rTMS (with any parameters) has a moderate, short-term antidepressant effect according to *N* = 40 RCTs (published between 1997–2008) and based on data from 1583 patients tested in 15 countries. This effect may not be secondary to treatment with antidepressants because it was observed in a subgroup of studies with unmedicated patients and treatment-resistant patients. Univariately, the short-term clinical efficacy of particularly the HFL rTMS may be better in female patients not controlling for any other study parameters. When adequate volume of data from primary RCTs becomes available, the future meta-analyses should focus on identifying the best combination of patient characteristics (demographic and clinical) and rTMS parameters that could further improve the short-term antidepressant response to rTMS.

## Additional file

Additional file 1:
**Methodological Appendix.**

## Abbreviations

AD: Antidepressant
B: Bilateral DLPFC
BDI: Beck Depression Inventory
BS: Bilateral sequential (left then right DLPFC)
C: Circular
*CI*: Confidence interval
*d*: Standardised mean difference (Cohen’s *d*)
D1: Antidepressants started on day 1
DLPFC: Dorsolateral prefrontal cortex
DSM-IV: Diagnostic and Statistical Manual of Mental Disorders
F3: The F3 location of the 10–20 electroencephalogram (EEG) system
F8: Figure-of-eight shape
Fig: Figure
HAMD: Hamilton Depression Rating Scale
HFL: High-frequency rTMS of the left DLPFC
ICD-10: International Statistical Classification of Diseases and Related Health Problems
L: Left DLPFC
LFR: Low-frequency rTMS of the right DLPFC
MADRS: Montgomery Åsberg Depression Rating Scale
MRI: Magnetic resonance imaging
*N*: Number of studies
N/A: Not reported or inadequate information
PRISMA: Preferred Reporting Items for Systematic Reviews and Meta-Analyses
R: Right DLPFC
RCT: Randomised-controlled trial
rTMS: Repetitive transcranial magnetic stimulation
*SD*: Standard deviation
Std diff: Standardized mean difference (Cohen’s *d*)
Tab: Table
TI: Tangential with inactive coil
TS: Tangential with sham coil containing embedded magnetic shield
