# Supplementary material for: Short-term efficacy of repetitive transcranial magnetic stimulation (rTMS) in depression- reanalysis of data from meta-analyses up to 2010
Source: BMC Psychol. 2014 Oct 7;2(1):39. doi: 10.1186/s40359-014-0039-y (PMC4317138; doi:10.1186/s40359-014-0039-y)
Supplement: Additional file 1: — Methodological Appendix. [file 40359_2014_39_MOESM1_ESM.pdf]

Additional file 1 (Methodological Appendix)

Kedzior, K, & Reitz, S (2014). Short-term efficacy of repetitive transcranial magnetic stimulation (rTMS) in depression- reanalysis of data from meta-analyses up to 2010. *BMC Psychology*.

Table of Contents

PRISMA checklist (Moher et al. 2009) 2

Mathematical approach used in the current meta-analysis 4

1. Combining of data from independent (active rTMS) subgroups within studies 4
2. Combining of data in dependent subgroups at different points in time (pre and post) 5
3. Effect size computation (standardised mean difference)- Cohen's *d* and Hedges' *g* 6
4. Combining multiple outcomes within studies 7
5. Meta-analysis: random-effects model with inverse-variance weights 7
6. Computation of R^2 in meta-regression 8

Table S1 Search strategy in the past 13 meta-analyses up to 2010 10

Table S2 List of 53 primary sources from the past 13 meta-analyses 11

Figure S1 Random-effects meta-analysis of *N*=40 studies 13

Figure S2 Cumulative random-effects meta-analysis of *N*=40 studies 14

Figure S3 One-study removed random-effects meta-analysis of *N*=40 studies 15

Figure S4 Funnel plot of the random-effects meta-analysis of *N*=40 studies 16

Figure S5 Random-effects meta-analysis of *N*=33 HFL studies with one outlier 17

Figure S6 Cumulative random-effects meta-analysis of *N*=32 HFL studies 19

Figure S7 One-study removed random-effects meta-analysis of *N*=32 HFL studies 20

Figure S8 Meta-regression of % female on weighted *d* in *N*=31 HFL studies 21

References 22

PRISMA checklist (Moher et al. 2009)

Section/topic	#	Checklist item	Reported on page #
**TITLE**			
Title	1	Identify the report as a systematic review, meta-analysis, or both.	1
**ABSTRACT**			
Structured summary	2	Provide a structured summary including, as applicable: background; objectives; data sources; study eligibility criteria, participants, and interventions; study appraisal and synthesis methods; results; limitations; conclusions and implications of key findings; systematic review registration number.	1
**INTRODUCTION**			
Rationale	3	Describe the rationale for the review in the context of what is already known.	1-3
Objectives	4	Provide an explicit statement of questions being addressed with reference to participants, interventions, comparisons, outcomes, and study design (PICOS).	2-3
**METHODS**			
Protocol and registration	5	Indicate if a review protocol exists, if and where it can be accessed (e.g., Web address), and, if available, provide registration information including registration number.	-
Eligibility criteria	6	Specify study characteristics (e.g., PICOS, length of follow-up) and report characteristics (e.g., years considered, language, publication status) used as criteria for eligibility, giving rationale.	3
Information sources	7	Describe all information sources (e.g., databases with dates of coverage, contact with study authors to identify additional studies) in the search and date last searched.	Tables S1-S2
Search	8	Present full electronic search strategy for at least one database, including any limits used, such that it could be repeated.	Table S1
Study selection	9	State the process for selecting studies (i.e., screening, eligibility, included in systematic review, and, if applicable, included in the meta-analysis).	3; Figure 1
Data collection process	10	Describe method of data extraction from reports (e.g., piloted forms, independently, in duplicate) and any processes for obtaining and confirming data from investigators.	3
Data items	11	List and define all variables for which data were sought (e.g., PICOS, funding sources) and any assumptions and simplifications made.	3; Tables 1-2
Risk of bias in individual studies	12	Describe methods used for assessing risk of bias of individual studies (including specification of whether this was done at the study or outcome level), and how this information is to be used in any data synthesis.	4; 10
Summary measures	13	State the principal summary measures (e.g., risk ratio, difference in means).	4

Synthesis of results	14	Describe the methods of handling data and combining results of studies, if done, including measures of consistency (e.g., I^2) for each meta-analysis.	4; 10
**Section/topic**	**#**	**Checklist item**	**Reported on page #**
Risk of bias across studies	15	Specify any assessment of risk of bias that may affect the cumulative evidence (e.g., publication bias, selective reporting within studies).	10
Additional analyses	16	Describe methods of additional analyses (e.g., sensitivity or subgroup analyses, meta-regression), if done, indicating which were pre-specified.	10
**RESULTS**			
Study selection	17	Give numbers of studies screened, assessed for eligibility, and included in the review, with reasons for exclusions at each stage, ideally with a flow diagram.	3-4; Figure 1, Table S2
Study characteristics	18	For each study, present characteristics for which data were extracted (e.g., study size, PICOS, follow-up period) and provide the citations.	Tables 1-2
Risk of bias within studies	19	Present data on risk of bias of each study and, if available, any outcome level assessment (see item 12).	Figures 2 S1-S8
Results of individual studies	20	For all outcomes considered (benefits or harms), present, for each study: (a) simple summary data for each intervention group (b) effect estimates and confidence intervals, ideally with a forest plot.	Figures 2, S1, S6
Synthesis of results	21	Present results of each meta-analysis done, including confidence intervals and measures of consistency.	10-11
Risk of bias across studies	22	Present results of any assessment of risk of bias across studies (see Item 15).	11; 13; Table 3; Figures 2, S4-S5
Additional analysis	23	Give results of additional analyses, if done (e.g., sensitivity or subgroup analyses, meta-regression [see Item 16]).	Figures 3, S2-S3, S6-S8
**DISCUSSION**			
Summary of evidence	24	Summarize the main findings including the strength of evidence for each main outcome; consider their relevance to key groups (e.g., healthcare providers, users, and policy makers).	11; 13-16
Limitations	25	Discuss limitations at study and outcome level (e.g., risk of bias), and at review-level (e.g., incomplete retrieval of identified research, reporting bias).	15-16
Conclusions	26	Provide a general interpretation of the results in the context of other evidence, and implications for future research.	11; 13-16
**FUNDING**			
Funding	27	Describe sources of funding for the systematic review and other support (e.g., supply of data); role of funders for the systematic review.	16

Mathematical approach used in the current meta-analysis

The approach to meta-analysis and all formulae in this document are based on the method of Hedges' et al. (Borenstein et al. 2009).

1. Combining of data from independent (active rTMS) subgroups within studies

Some studies in the current meta-analysis compared one sham group with more than one active rTMS groups. Thus, for the purposes of the overall meta-analysis the data from the multiple active subgroups were combined into one active rTMS group to compute only one effect size for the study. Using one example study (Padberg et al. 1999), the mean *(M)* and standard deviation *(SD)* of depression scores for the two active subgroups (stimulation frequencies of either 10 Hz or .3 Hz) were combined into one group at each of the two points in time (pre and post-treatment) according to the following formulae (Borenstein et al. 2009 p. 222):

- The combined mean depression score for the 'active-pre' group (M_{1+2}) was computed by weighing the mean depression score of subgroup 1 (M_1; 10 Hz) and subgroup 2 (M_2; .3 Hz) based on the sample size of each subgroup (N_1 and N_2):

$$M_{1+2} = \frac{N_1 M_1 + N_2 M_2}{N_1 + N_2}$$

  The combined mean depression score for the other group ('active-post') was computed the same way.

- The combined standard deviation of the mean depression scores for the 'active-pre' group (SD_{1+2}) was computed using individual *SD* and *N* values of subgroup 1 (10 Hz; SD_1 and N_1) and subgroup 2 (.3 Hz; SD_2 and N_2):

$$SD_{1+2} = \sqrt{\frac{(N_1 - 1)SD_1^{\,2} + (N_2 - 1)SD_2^{\,2} + \frac{N_1 N_2}{N_1 + N_2}(M_1 - M_2)^2}{N_1 + N_2 - 1}}$$

  The combined standard deviation of the mean depression scores for the other group ('active-post') was computed the same way.

- The combined sample size for the 'active-pre' group (N_{1+2}) was computed by adding the sample sizes of the two subgroups (N_1 + N_2). The combined sample size for the other group ('active-post') was computed the same way.

The study by Stern and colleagues (Stern et al. 2007), was performed on three active subgroups (10 Hz left DLPFC, 1 Hz left DLPFC, and 1 Hz right DLPFC). Thus, the two left-stimulation subgroups (10 Hz and 1 Hz) were combined first and these (combined) scores were then combined with the scores of the right-stimulation subgroup according to the formulae described above.

2. Combining of data in dependent subgroups at different points in time (pre and post)

Since data were collected from the same groups (sham or active) twice (pre and post treatment) it was necessary to reduce them to one score/group for the purposes of meta-analysis. Following the approach of Holtzheimer and colleagues (Holtzheimer et al. 2001), such reduction in scores was performed by expressing the severity of depression scores as difference scores: mean depression at baseline (pre-treatment) – last session (post-treatment) in each group separately (M_S in sham or M_A in active groups).

The total sample size N of each group (N_S or N_A) was either the sample size at baseline or the mean sample size at baseline and last session if any patients dropped out of the study.

The SD of the mean difference scores was computed for each group separately (SD_S or SD_A) as follows (Borenstein et al. 2009 p. 234):

$$SD = \sqrt{SD_{pre}^2 + SD_{post}^2 - 2rSD_{pre}SD_{post}}$$

The correlation coefficient r=.5, between the pre- and the post-treatment depression scores, was chosen as the most optimal coefficient that neither overestimates the SD (r=.0) nor underestimates the SD (r=1.0) (Borenstein et al. 2009 p. 237, Table 24.7). The value of .5 was also close to the mean of correlations between pre- and post-treatment scores conducted in studies that reported the scores for all individual patients (these studies are listed in the table below). Specifically, the correlation coefficients were r=.37 (all patients), r=.38 (sham), and r=.49 (active group).

Table. Pearson correlation coefficients *r* between mean severity of depression scores at baseline (pre) and after the last session (post) of rTMS (or sham) in studies that reported such scores for individual patients

		Study by first author and year	r_both	N_both	r_sham	N_sham	r_active	N_active
1		Berman 2000 HAMD25	.28	20	.48	10	.20	10
2		Boutros 2002 HAMD25	.50	18	.02	7	.67	11
3		George 1997 HAMD21	.22	12	.06	5	.70	7
4		Kimbrell 1999 HAMD21	.48	13	.97	3	.38	10
Total	N	4	4	4	4	4	4	4
	Mean		.3700	15.75	.3825	6.25	.4875	9.50
	Sum		1.48	63	1.53	25	1.95	38

Notes: All studies reported the scores on HAMD (different versions listed in the table). Abbreviations: HAMD, Hamilton Depression Rating Scale; rTMS, repetitive transcranial magnetic stimulation.

The *M* and *SD* of the difference scores and the total *N* per group (sham and active) were used to compute the effect sizes.

3. Effect size computation (standardised mean difference)- Cohen's *d* and Hedges' *g*

The standardised mean difference, Cohen's *d*, was computed for the sham and active rTMS groups in each study as follows (Borenstein et al. 2009 p. 26):

$$d = \frac{M_S - M_A}{SD_{pooled}} \qquad SD_{pooled} = \sqrt{\frac{(N_S - 1)SD_S^{\ 2} + (N_A - 1)SD_A^{\ 2}}{N_S + N_A - 2}},$$

where M_S and M_A refer to the mean severity of depression difference score (pre-post) in sham and active rTMS groups respectively, SD_{pooled} is the *SD* of the severity of depression difference score (pre-post) pooled for the two groups using the standard deviations of sham (SD_S) and active rTMS (SD_A) groups, and N_S and N_A are the sizes of sham and active groups respectively. The variance of *d* (V_d) was computed as follows (Borenstein et al. 2009 p. 27):

$$V_d = SD_d^2 = \frac{N_S + N_A}{N_S N_A} + \frac{d^2}{2(N_S + N_A)}$$

In addition to *d*, the standardised mean difference, Hedges' *g* (that is an unbiased version of *d* in small-*N* studies) and its variance (V_g) were computed for the sham and active stimulation groups in each study as follows (Borenstein et al. 2009 p. 27):

$$g = d \times J = d(1 - \frac{3}{4(N_S + N_A) - 9}) \qquad V_g = V_d \times J^2,$$

where *J* is the correction factor.

4. Combining multiple outcomes within studies

Some studies in the current analysis utilised multiple scales to measure depression severity (HAMD, BDI, and MADRS). In such cases, the effect sizes *d* and their variance (V_d) were computed separately for each scale. Subsequently, one mean effect size *d* was computed/study using an arithmetic mean. The variance of such a mean effect size (V_{dmean}) was computed according to the following formula for combining multiple outcomes within the same studies (Borenstein et al. 2009 p. 227):

$$V_{d_{mean}} = \frac{1}{4} \times \left(V_{d1} + V_{d2} + 2r\sqrt{V_{d1}}\sqrt{V_{d2}} \right)$$

, where r=1.0 (correlation coefficient between outcomes in the same cases). Subsequent meta-analysis was computed on such a mean effect size of multiple outcomes/study and its variance.

5. Meta-analysis: random-effects model with inverse-variance weights

The weight in each study (W_d) was computed according to the random-effects model as follows (Borenstein et al. 2009 p. 73):

$$W_d = \frac{1}{V_d + T^2},$$

where V_d is the within-study variance (variance of *d)* and T_2 is the between-study variance which was computed according to the method of moments (or the DerSimonian and Laird method (DerSimonian and Laird 1986)) and using $df=k-1$ (k=number of studies) as follows (Borenstein et al. 2009 p. 73-74):

$$T^2 = \frac{Q - df}{C} \qquad C = \sum \frac{1}{V_d} - \frac{\sum \left(\frac{1}{V_d} \right)^2}{\sum \frac{1}{V_d}} \qquad Q = \sum \frac{(d - M_d)^2}{V_d}$$

The overall mean weighted effect size (M_d) and its variance (V_{Md}) were computed for subgroups of studies as follows (Borenstein et al. 2009 p. 73-74):

$$M_d = \frac{\sum W_d \times d}{\sum W_d} \qquad V_{M_d} = \frac{1}{\sum W_d}$$

The lower and upper 95% confidence intervals of M_d (LCI_{Md} and UCI_{Md}) were computed as follows (Borenstein et al. 2009 p. 73-74):

$$LCI_{M_d} = M_d - 1.96 \times \sqrt{V_{M_d}} \qquad UCI_{M_d} = M_d + 1.96 \times \sqrt{V_{M_d}}$$

Finally, the *z*-score for M_d was computed, to test the null-hypothesis that M_d=0 meaning that rTMS is not effective at reducing depression scores compared to sham, according to the following formula (Borenstein et al. 2009 p. 74):

$$Z = \frac{M_d}{\sqrt{V_{M_d}}}$$

6. Computation of R^2 in meta-regression

Univariate linear meta-regressions were computed using the random-effects model to find out if the weighted effect sizes (outcome) could be predicted using the various study characteristics (clinical, demographic, and the rTMS parameters) in the current meta-analysis. The slope of the straight line (the line of best fit), *B**, was tested for statistical significance according to the following formula (Borenstein et al. 2009 p. 197):

$$Z^* = \frac{B^*}{SE_{B*}}$$

The null-hypothesis tested was that *B** is not different from zero (meaning that the predictor does not predict the outcome). Since univariate regressions were conducted (using one predictor only), it was assumed that the statistical significance of the slope of the regression line was equivalent to the statistical significance of the regression model.

The practical significance of the statistically significant regression models was tested using the equivalent of the R^2 index in linear regression. The formula for R^2 in meta-regression takes into account the between-study variance in the weighted *d* unexplained by the regression model containing the predictor (T^2_{model} shown as ‘Tau-squared’ in the output of the meta-regression module in CMA) and the total within- and between-study variance among the weighted *d* (T^2_{total} that is computed together with other heterogeneity

statistics in the standard random-effects model of all studies involved in the meta-regression) as follows (Borenstein et al. 2009 p. 202):

$$R^2 = 1 - \frac{T^2{}_{model}}{T^2{}_{total}}$$

The R^2 in meta-regression shows the proportion of the between-study variance in weighted *d* explained by the predictor.

Table S1 Search strategy in the past 13 meta-analyses up to 2010

Study	Database (Time frame)	Key words (search limits)	*N* studies
(Dell'Osso et al. 2011)	PubMed (Jan 1980-Dec 2010)	Transcranial magnetic stimulation; TMS; rTMS; depression; major depression; depressive disorders; treatment resistant depression; dysthymic disorder (English)	13 meta-analyses
1. (McNamara et al. 2001)	Medline (1966- Jan 2000), Embase/Excerpta Medica (1980- Jan 2000), Biological Abstracts and Index of Scientific and Technical Proceedings, Meta-Register of Controlled Trials (www.controlled-trials.com), the National Register (www.doh.gov.uk/research/ntr), Cochrane Library, Omni Biomedical Search Tool (www.omni.ac.uk), TMS Resources and Published Articles (www.musc.edu/tmsmirror/TMSresrc), Avery-George Index (www.ists.unibe.ch/ists/TMSAvery), the International Society of Transcranial Magnetic Stimulation (www.ists.unibe.ch/), TMS and Depression (www.psycom.net/depression.central.transcranial), the Helsinki TMS (www.biomag.helsinki.fi/tms/)	–	*N*=5
2. (Holtzheimer et al. 2001)	Medline, International Society of Transcranial Magnetic Stimulation (www.ists.unibe.ch/)	transcranial magnetic stimulation; TMS; rTMS; depression	*N*=12
3. (Rodriguez-Martin et al. 2001)*	Cochrane Collaboration Depression, Anxiety and Neurosis Review Group trials register (<June 2001), the Cochrane Controlled Trials Register (Issue 2, 2001), Medline (1966-2001), Embase (1974-2001), PsycLit (1980-2001)	–	*N*=16
4. (Kozel and George 2002)	PsycInfo (1887- April 2002), Medline (1966-April 2002), Current Contents (April 2001- April 2002), meta-analyses (Burt et al. 2002; McNamara et al. 2001)	TMS; rTMS; transcranial magnetic stimulat: and depression or depressive disorder (English)	*N*=12
5. (Burt et al. 2002)	–	–	*N*=16
6. (Martin et al. 2003)	Medline (1966-March 2002), Embase (1974- March 2002), PsycLit (1980-2001), the Register of Clinical Trials of the Cochrane Collaboration Depression, Neurosis and Anxiety Review Group (January 2002), Cochrane Controlled Trials Register (January 2002)	magnetic-stimulation; TMS; rTMS; depression; depressive disorder; dysthmic disorder	*N*=14
7. (Couturier 2005)	Medline, Cochrane Database of Controlled Trials, metaRegister of Controlled Trials (www.controlled-trials.com/mrct), abstracts from scientific meetings: the Society of Biological Psychiatry (2002-2003), the American Psychiatric Association (2000-2003)	transcranial magnetic stimulation; transcranial magnetic stimulation AND depression (review; randomised controlled trial)	*N*=6
8. (Herrmann and Ebmeier 2006)	Medline, Embase, Cochrane Database of Controlled Trials	transcranial magnetic stimulation; TMS; depression	*N*=33
9. (Gross et al. 2007)	– (December 2005- November 2006)	major depression; depression; transcranial magnetic stimulation; rTMS; TMS	*N*=5
10. (Lam et al. 2008)	Medline (>1966), Embase (>1980), PsycInfo (>1974), Cochrane Central Register of Controlled Trials databases (<May 15, 2008)	transcranial; magnetic; depress*	*N*=24
11. (Schutter 2009)	PubMed, Web of Science (January 1980-November 2007)	depression; transcranial magnetic stimulation	*N*=30
12. (Schutter 2010)	PubMed, Web of Science (January 1994-July 2009)	depression; transcranial magnetic stimulation	*N*=9
13. (Slotema et al. 2010)	PubMed (1990- October 2008), Medline (1990- October 2008), Embase Psychiatry (1997- October 2008), Cochrane Central Register of Controlled Trials, Cochrane Database of Systematic Reviews, Database of Abstracts of Reviews of Effects, PsycINFO (1990-October 2008)	transcranial magnetic stimulation; TMS; repetitive TMS; psychiatry; mental disorder; psychiatric disorder; bipolar disorder; depression	*N*=34

Table S2 List of 53 primary sources from the past 13 meta-analyses

	Year	Study (by year and first author)	13 meta-analyses												
			1. (McNamara et al. 2001)	2. (Holtzheimer et al. 2001)	3. (Rodriguez-Martin et al. 2001)	4. (Kozel and George 2002)	5. (Burt et al. 2002)	6. (Martin et al. 2003)	7. (Couturier 2005)	8. (Herrmann and Ebmeier 2006)	9. (Gross et al. 2007)	10. (Lam et al. 2008)	11. (Schutter 2009)	12. (Schutter 2010)	13. (Slotema et al. 2010)
		**TOTAL *N* STUDIES**	**5**	**13**	**16**	**12**	**16**	**14**	**6**	**33**	**5**	**24**	**30**	**9**	**34**
1	1995	(Kolbinger et al. 1995)*			+		+							+	
2	1996	(Conca et al. 1996)*			+		+								
3		(Pascual-Leone et al. 1996)*	+	+			+			+					
4	1997	(George et al. 1997)	+	+	+	+	+	+	+	+			+		
5		(Haag et al. 1997)*			+								+		
6	1999	(Avery et al. 1999)		+	+	+	+	+	+	+			+		+
7		(Kimbrell et al. 1999)		+	+	+	+	+	+	+			+		
8		(Klein et al. 1999)	+	+	+		+	+		+				+	+
9		(Loo et al. 1999)		+	+	+	+	+		+			+		+
10		(Padberg et al. 1999)	+	+	+	+	+	+	+	+		+	+	+	+
11		(Stikhina et al. 1999)*					+			+					
12	2000	(Avery et al. 2000)*		+											
13		(Berman et al. 2000)	+	+	+	+	+	+		+		+	+		+
14		(Eschweiler et al. 2000)		+	+	+	+	+	+	+			+		
15		(George et al. 2000)		+	+	+	+	+	+	+			+		+
16		(Grunhaus et al. 2000)*			+										
17	2001	(Garcia-Toro et al. 2001)		+	+	+	+	+				+	+		+
18		(García-Toro et al. 2001)			+	+		+		+		+			+
19		(Lisanby et al. 2001)*				+	+			+					
20		(Manes et al. 2001)				+	+	+		+		+	+		+
21		(Szuba et al. 2001)						+					+		
22	2002	(Boutros et al. 2002)								+		+	+		+
23		(Padberg et al. 2002)								+		+	+		
24	2003	(Fitzgerald et al. 2003)								+		+	+	+	+
25		(Herwig et al. 2003)								+					+
26		(Höppner et al. 2003)								+			+	+	+
27		(Loo et al. 2003)								+		+			+
28		(Nahas et al. 2003)								+			+		
29	2004	(Buchholtz et al. 2004)													+
30		(Hausmann et al. 2004)								+					+
31		(Holtzheimer et al. 2004)		+						+			+		+
32		(Jorge et al. 2004)*								+			+		
33		(Kauffmann et al. 2004)								+		+		+	+
34		(Koerselman et al. 2004)								+			+		+
35		(Mosimann et al. 2004)			+			+		+		+	+		+
36		(Poulet et al. 2004)								+			+		+
37	2005	(Miniussi et al. 2005)*								+		+	+	+	
38		(Rossini et al. 2005)									+				+
39		(Rossini et al. 2005)*								+		+	+		
40		(Rumi et al. 2005)								+			+		
41		(Su et al. 2005)										+			+
42	2006	(Avery et al. 2006)								+	+	+	+		
43		(Fitzgerald et al. 2006)									+	+			+
44		(Garcia-Toro et al. 2006)									+	+			+
45		(Januel et al. 2006)								+	+			+	+
46		(Moller et al. 2006)										+			
47	2007	(Anderson et al. 2007)										+			+
48		(Bortolomasi et al. 2007)													+
49		(Herwig et al. 2007)											+		+
50		(Loo et al. 2007)										+	+		+
51		(O'Reardon et al. 2007)										+	+		+
52		(Stern et al. 2007)										+		+	+
53	2008	(Bretlau et al. 2008)										+			
54		(Fitzgerald et al. 2008)*													+
55		(Mogg et al. 2008)*										+	+		+

Notes: There were 55 sources in total because data from two abstracts excluded from the current analysis ((Haag et al. 1997) and (Avery et al. 2000)) were later included in published articles included in the current analysis ((Padberg et al. 1999) and (Holtzheimer et al. 2004)).

*Studies excluded from the current analysis (13/53 or 24% studies excluded). The exclusion criteria were:

- *N*=1: DLPFC not stimulated (vertex, Cz location, stimulated) (Kolbinger et al. 1995)
- *N*=3: No sham group (Conca et al. 1996; Fitzgerald et al. 2008; Grunhaus et al. 2000)
- *N*=2: Sham at location other than DLPFC: parietal cortex (active stimulation) (Herwig et al. 2003), occipital cortex (active stimulation, coil tilted at 45°) (Moller et al. 2006)
- *N*=1: Cross-over design used (Pascual-Leone et al. 1996)
- *N*=5: Inadequate data reported and/or lack of response from authors:
    - baseline scores for individual groups and after treatment scores missing (Lisanby et al. 2001)
    - parallel stimulation applied only after all participants received active stimulation and *SD* values for HAMD scores missing (Miniussi et al. 2005)
    - *SD* values for HAMD scores missing (Rossini et al. 2005; Stikhina et al. 1999)
    - after treatment HAMD28 scores missing (Szuba et al. 2001)
- *N*=1: Depression secondary to stroke and *SD* values for HAMD scores missing (Jorge et al. 2004)

Figure S1 Random-effects meta-analysis of *N*=40 studies

Study name	Outcome	Statistics for each study: Std diff in means	Lower limit	Upper limit	p-Value	Sample size: Sham	rTMS
George et al. (1997)	HAMD	-1.44	-2.73	-0.16	0.028	5	7
Avery et al. (1999)	Combined	-0.50	-2.23	1.23	0.572	2	4
Kimbrell et al. (1999) all	HAMD	-0.44	-1.75	0.86	0.503	3	10
Klein et al. (1999)	Combined	-0.75	-1.24	-0.25	0.003	32	35
Loo et al. (1999)	Combined	0.25	-0.67	1.18	0.592	9	9
Padberg et al. (1999) all	HAMD	-0.58	-1.57	0.42	0.258	6	12
Berman et al. (2000)	HAMD	-1.21	-2.17	-0.26	0.013	10	10
Eschweiler et al. (2000)	Combined	-1.35	-2.62	-0.08	0.038	5	7
George et al. (2000) all	HAMD	-0.70	-1.48	0.08	0.080	10	20
Garcia-Toro et al. (2001a)	Combined	-0.63	-1.31	0.05	0.071	18	17
Garcia-Toro et al. (2001b)	Combined	-0.44	-1.29	0.41	0.309	11	11
Manes et al. (2001)	HAMD	-0.30	-1.18	0.58	0.505	10	10
Boutros et al. (2002)	HAMD	-0.25	-1.20	0.70	0.606	7	11
Padberg et al. (2002)	Combined	-0.99	-1.92	-0.06	0.038	10	10
Fitzgerald et al. (2003) all	Combined	-0.44	-0.99	0.10	0.110	20	40
Höppner et al. (2003)	Combined	0.19	-0.70	1.08	0.676	10	10
Loo et al. (2003)	Combined	-0.12	-1.03	0.78	0.787	10	9
Nahas et al. (2003)	HAMD	0.09	-0.73	0.91	0.828	12	11
Buchholtz et al. (2004)	HAMD	0.14	-0.95	1.24	0.796	7	6
Hausmann et al. (2004)	Combined	-0.34	-1.02	0.33	0.318	13	25
Holtzheimer et al. (2004)	Combined	-0.22	-1.24	0.80	0.674	8	7
Kauffmann et al. (2004)	HAMD	-0.84	-2.03	0.36	0.171	5	7
Koerselman et al. (2004)	HAMD	-0.15	-0.70	0.40	0.584	25	26
Mosimann et al. (2004)	Combined	-0.12	-0.95	0.71	0.774	9	15
Poulet et al. (2004)	Combined	0.13	-0.77	1.04	0.772	9	10
Rossini et al. (2005)	HAMD	-0.71	-1.13	-0.30	0.001	47	49
Rumi et al. (2005)	MADRS	-1.23	-1.87	-0.60	0.000	24	22
Su et al. (2005) all	Combined	-1.14	-1.95	-0.33	0.006	10	20
Avery et al. (2006)	Combined	-0.67	-1.16	-0.18	0.007	33	35
Fitzgerald et al. (2006)	Combined	-0.53	-1.11	0.05	0.075	22	25
Garcia-Toro et al. (2006)	HAMD	-0.80	-1.71	0.11	0.086	10	10
Januel et al. (2006)	HAMD	-1.58	-2.46	-0.71	0.000	16	11
Anderson et al. (2007)	MADRS	-0.78	-1.60	0.04	0.063	14	11
Bortolomasi et al. (2007)	Combined	-1.20	-2.20	-0.19	0.020	7	12
Herwig et al. (2007)	Combined	-0.17	-0.54	0.19	0.355	59	57
Loo et al. (2007)	Combined	-0.34	-0.98	0.30	0.298	19	19
OReardon et al. (2007)	Combined	-0.30	-0.53	-0.08	0.009	146	155
Stern et al. (2007) all	HAMD	-1.45	-2.16	-0.74	0.000	14	29
Bretlau et al. (2008)	HAMD	-0.75	-1.35	-0.15	0.015	23	22
Mogg et al. (2008)	Combined	-0.46	-0.99	0.07	0.087	29	28
		-0.54	-0.68	-0.41	0.000		

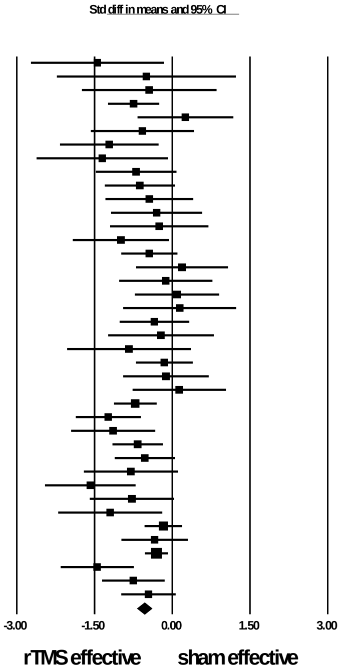

Notes: The data shown on this figure are also included in the meta-analysis of *N*=54 studies (*N*=40 studies from this analysis and *N*=14 new studies) shown on Figure S3 (Kedzior et al. 2014). 'All' refers to all patients in multiple active rTMS groups who received rTMS with different parameters. 'Combined' in the column 'Outcome' indicates that more than one depression scale was used in a study and the effect sizes according to the multiple scales were combined into one. The mean number of patients per group was used in the final calculations if patients dropped out throughout the study between baseline and final sessions. The forest plot shows the weighted effect size *d* (box) and its 95% *CI* (vertical line through the box) for each study in the analysis. The diamond depicts the overall mean weighted *d* of all studies and its 95% *CI* (width of the diamond). The mean depression scores (baseline – final) were significantly reduced after rTMS compared to sham in 40 studies (overall mean weighted *d*=−.54, 95% *CI*: −.68, −.41). Abbreviations: *CI*, confidence interval; HAMD, Hamilton Depression Rating Scale; MADRS, Montgomery Åsberg Depression Rating Scale; rTMS, repetitive transcranial magnetic stimulation; Std diff, standardized mean difference (Cohen's *d*).

Figure S2 Cumulative random-effects meta-analysis of *N*=40 studies

Study name	Outcome	Cumulative statistics: Point	Lower limit	Upper limit	p-Value	Sham	rTMS
George et al. (1997)	HAMD	-1.44	-2.73	-0.16	0.028	5	7
Avery et al. (1999)	Combined	-1.11	-2.14	-0.08	0.035	7	11
Kimbrell et al. (1999) all	HAMD	-0.85	-1.66	-0.04	0.039	10	21
Klein et al. (1999)	Combined	-0.77	-1.20	-0.35	0.000	42	56
Loo et al. (1999)	Combined	-0.56	-1.07	-0.05	0.030	51	65
Padberg et al. (1999) all	HAMD	-0.58	-0.97	-0.20	0.003	57	77
Berman et al. (2000)	HAMD	-0.67	-1.04	-0.29	0.000	67	87
Eschweiler et al. (2000)	Combined	-0.72	-1.07	-0.36	0.000	72	94
George et al. (2000) all	HAMD	-0.71	-1.01	-0.41	0.000	82	114
Garcia-Toro et al. (2001a)	Combined	-0.70	-0.97	-0.42	0.000	100	131
Garcia-Toro et al. (2001b)	Combined	-0.67	-0.94	-0.41	0.000	111	142
Manes et al. (2001)	HAMD	-0.64	-0.90	-0.39	0.000	121	152
Boutros et al. (2002)	HAMD	-0.62	-0.86	-0.38	0.000	128	163
Padberg et al. (2002)	Combined	-0.64	-0.88	-0.41	0.000	138	173
Fitzgerald et al. (2003) all	Combined	-0.61	-0.83	-0.40	0.000	158	213
Höppner et al. (2003)	Combined	-0.57	-0.78	-0.36	0.000	168	223
Loo et al. (2003)	Combined	-0.54	-0.75	-0.34	0.000	178	232
Nahas et al. (2003)	HAMD	-0.51	-0.70	-0.31	0.000	190	243
Buchholtz et al. (2004)	HAMD	-0.49	-0.68	-0.29	0.000	197	249
Hausmann et al. (2004)	Combined	-0.48	-0.66	-0.29	0.000	210	274
Holtzheimer et al. (2004)	Combined	-0.47	-0.65	-0.28	0.000	218	281
Kauffmann et al. (2004)	HAMD	-0.48	-0.66	-0.29	0.000	223	288
Koerselman et al. (2004)	HAMD	-0.44	-0.62	-0.27	0.000	248	314
Mosimann et al. (2004)	Combined	-0.43	-0.60	-0.26	0.000	257	329
Poulet et al. (2004)	Combined	-0.41	-0.58	-0.25	0.000	266	339
Rossini et al. (2005)	HAMD	-0.45	-0.61	-0.30	0.000	313	388
Rumi et al. (2005)	MADRS	-0.49	-0.65	-0.33	0.000	337	410
Su et al. (2005) all	Combined	-0.51	-0.67	-0.35	0.000	347	430
Avery et al. (2006)	Combined	-0.52	-0.68	-0.37	0.000	380	465
Fitzgerald et al. (2006)	Combined	-0.52	-0.67	-0.38	0.000	402	490
Garcia-Toro et al. (2006)	HAMD	-0.53	-0.67	-0.39	0.000	412	500
Januel et al. (2006)	HAMD	-0.55	-0.71	-0.40	0.000	428	511
Anderson et al. (2007)	MADRS	-0.56	-0.71	-0.41	0.000	442	522
Bortolomasi et al. (2007)	Combined	-0.57	-0.72	-0.43	0.000	449	534
Herwig et al. (2007)	Combined	-0.54	-0.69	-0.40	0.000	508	591
Loo et al. (2007)	Combined	-0.53	-0.67	-0.39	0.000	527	610
OReardon et al. (2007)	Combined	-0.51	-0.64	-0.38	0.000	673	765
Stern et al. (2007) all	HAMD	-0.54	-0.68	-0.40	0.000	687	794
Bretlau et al. (2008)	HAMD	-0.55	-0.69	-0.41	0.000	710	816
Mogg et al. (2008)	Combined	-0.54	-0.68	-0.41	0.000	739	844
		-0.54	-0.68	-0.41	0.000		

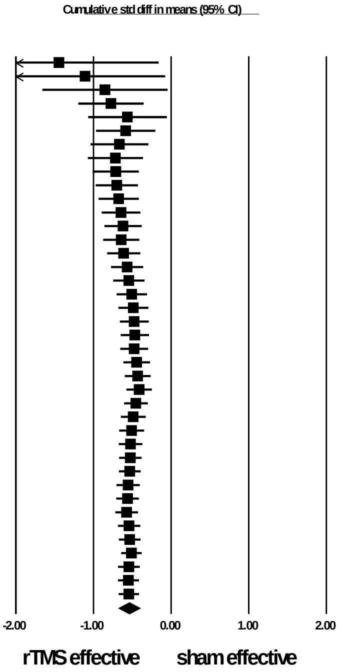

Notes: The data shown on this figure are also included in the cumulative analysis of *N*=54 studies (*N*=40 studies from this analysis and *N*=14 new studies) shown on Figure S5 (Kedzior et al. 2014). 'All' refers to all patients in multiple active rTMS groups who received rTMS with different parameters. 'Combined' in the column 'Outcome' indicates that more than one depression scale was used in a study and the effect sizes according to the multiple scales were combined into one. 'Point' refers to the overall mean weighted *d* of all studies before and including the study listed in each row. Abbreviations: *CI*, confidence interval; HAMD, Hamilton Depression Rating Scale; MADRS, Montgomery Åsberg Depression Rating Scale; rTMS, repetitive transcranial magnetic stimulation; Std diff, standardized mean difference (Cohen's *d*).

Figure S3 One-study removed random-effects meta-analysis of *N*=40 studies

Study name	Outcome	Statistics with study removed			
		Point	Lower limit	Upper limit	p-Value
George et al. (1997)	HAMD	-0.54	-0.67	-0.40	0.000
Avery et al. (1999)	Combined	-0.55	-0.68	-0.41	0.000
Kimbrell et al. (1999) all	HAMD	-0.55	-0.68	-0.41	0.000
Klein et al. (1999)	Combined	-0.54	-0.67	-0.40	0.000
Loo et al. (1999)	Combined	-0.56	-0.69	-0.43	0.000
Padberg et al. (1999) all	HAMD	-0.55	-0.68	-0.41	0.000
Berman et al. (2000)	HAMD	-0.53	-0.66	-0.40	0.000
Eschweiler et al. (2000)	Combined	-0.54	-0.67	-0.40	0.000
George et al. (2000) all	HAMD	-0.54	-0.68	-0.41	0.000
Garcia-Toro et al. (2001a)	Combined	-0.54	-0.68	-0.41	0.000
Garcia-Toro et al. (2001b)	Combined	-0.55	-0.68	-0.41	0.000
Manes et al. (2001)	HAMD	-0.55	-0.69	-0.42	0.000
Boutros et al. (2002)	HAMD	-0.55	-0.69	-0.42	0.000
Padberg et al. (2002)	Combined	-0.54	-0.67	-0.40	0.000
Fitzgerald et al. (2003) all	Combined	-0.55	-0.69	-0.41	0.000
Höppner et al. (2003)	Combined	-0.56	-0.69	-0.43	0.000
Loo et al. (2003)	Combined	-0.55	-0.69	-0.42	0.000
Nahas et al. (2003)	HAMD	-0.56	-0.69	-0.42	0.000
Buchholtz et al. (2004)	HAMD	-0.55	-0.69	-0.42	0.000
Hausmann et al. (2004)	Combined	-0.55	-0.69	-0.42	0.000
Holtzheimer et al. (2004)	Combined	-0.55	-0.68	-0.42	0.000
Kauffmann et al. (2004)	HAMD	-0.54	-0.68	-0.41	0.000
Koerselman et al. (2004)	HAMD	-0.56	-0.69	-0.43	0.000
Mosimann et al. (2004)	Combined	-0.55	-0.69	-0.42	0.000
Poulet et al. (2004)	Combined	-0.56	-0.69	-0.42	0.000
Rossini et al. (2005)	HAMD	-0.54	-0.67	-0.40	0.000
Rumi et al. (2005)	MADRS	-0.52	-0.65	-0.39	0.000
Su et al. (2005) all	Combined	-0.53	-0.66	-0.40	0.000
Avery et al. (2006)	Combined	-0.54	-0.68	-0.40	0.000
Fitzgerald et al. (2006)	Combined	-0.55	-0.68	-0.41	0.000
Garcia-Toro et al. (2006)	HAMD	-0.54	-0.68	-0.41	0.000
Januel et al. (2006)	HAMD	-0.52	-0.65	-0.39	0.000
Anderson et al. (2007)	MADRS	-0.54	-0.68	-0.41	0.000
Bortolomasi et al. (2007)	Combined	-0.53	-0.67	-0.40	0.000
Herwig et al. (2007)	Combined	-0.57	-0.70	-0.43	0.000
Loo et al. (2007)	Combined	-0.55	-0.69	-0.42	0.000
OReardon et al. (2007)	Combined	-0.57	-0.70	-0.43	0.000
Stern et al. (2007) all	HAMD	-0.51	-0.64	-0.39	0.000
Bretlau et al. (2008)	HAMD	-0.54	-0.67	-0.40	0.000
Mogg et al. (2008)	Combined	-0.55	-0.69	-0.41	0.000
		-0.54	-0.68	-0.41	0.000

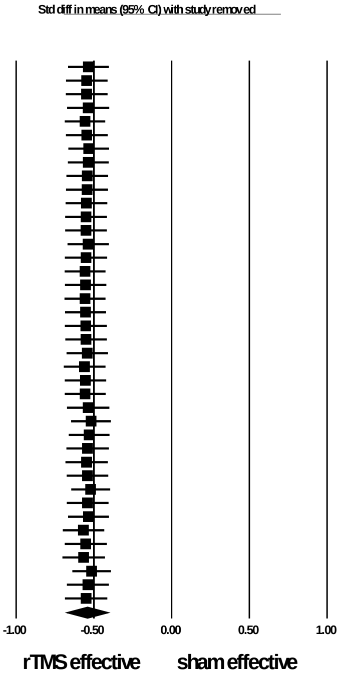

Notes: The data shown on this figure are also included in the one-study removed analysis of *N*=54 studies (*N*=40 studies from this analysis and *N*=14 new studies) shown on Figure S4 (Kedzior et al. 2014). 'All' refers to all patients in multiple active rTMS groups who received rTMS with different parameters. 'Combined' in the column 'Outcome' indicates that more than one depression scale was used in a study and the effect sizes according to the multiple scales were combined into one. 'Point' refers to the overall mean weighted *d* of all studies except the study listed in each row. Abbreviations: *CI*, confidence interval; HAMD, Hamilton Depression Rating Scale; MADRS, Montgomery Åsberg Depression Rating Scale; rTMS, repetitive transcranial magnetic stimulation; Std diff, standardized mean difference (Cohen's *d*).

Figure S4 Funnel plot of the random-effects meta-analysis of *N*=40 studies

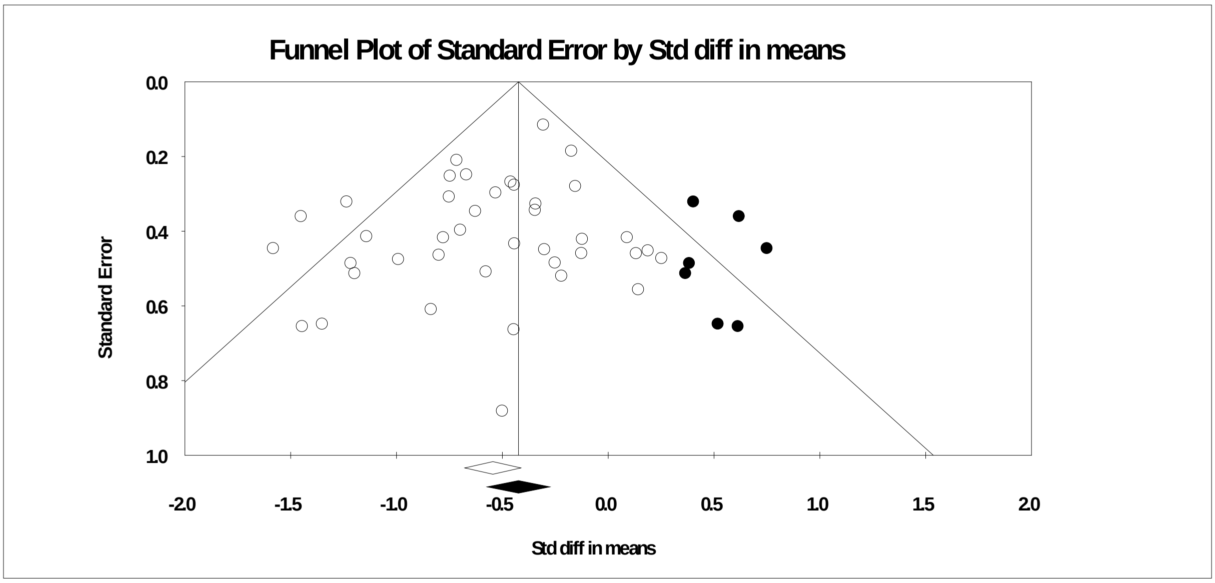

Notes: The funnel plot shows the effect size *d* versus standard error for each study in the analysis. The plot was asymmetrical around the overall mean weighted *d* and *N*=7 studies (filled circles) were theoretically missing from the analysis. The overall mean weighted effect size corrected for these seven studies indicated that antidepressant effect was still present in the data favouring rTMS over sham (corrected overall mean weighted *d*= -.42, *95%CI*: -.57, -.28). Thus, there was little evidence that publication bias affected the results of the current meta-analysis. Abbreviations: *CI*, confidence interval; rTMS, repetitive transcranial magnetic stimulation; Std diff, standardized mean difference (Cohen's *d*).

Figure S5 Random-effects meta-analysis of *N*=33 HFL studies with one outlier

Studyname	Outcome	Std diff in means	Lower limit	Upper limit	p-Value	Std Residual	P-Val
George et al. (1997)	HAMD	-1.44	-2.73	-0.16	0.028	-1.29	0.198
Avery et al. (1999)	Combined	-0.50	-2.23	1.23	0.572	0.03	0.978
Kimbrell et al. (1999)	HAMD	0.24	-1.20	1.68	0.744	0.97	0.331
Loo et al. (1999)	Combined	0.25	-0.67	1.18	0.592	1.41	0.158
Padberg et al. (1999)	HAMD	-0.40	-1.54	0.74	0.493	0.19	0.848
Berman et al. (2000)	HAMD	-1.21	-2.17	-0.26	0.013	-1.23	0.220
Eschweiler et al. (2000)	Combined	-1.35	-2.62	-0.08	0.038	-1.17	0.244
George et al. (2000) all	HAMD	-0.70	-1.48	0.08	0.080	-0.35	0.723
Garcia-Toro et al. (2001a)	Combined	-0.63	-1.31	0.05	0.071	-0.23	0.820
Garcia-Toro et al. (2001b)	Combined	-0.44	-1.29	0.41	0.309	0.16	0.873
Manes et al. (2001)	HAMD	-0.30	-1.18	0.58	0.505	0.42	0.673
Boutros et al. (2002)	HAMD	-0.25	-1.20	0.70	0.606	0.49	0.626
Padberg et al. (2002)	Combined	-0.99	-1.92	-0.06	0.038	-0.84	0.400
Fitzgerald et al. (2003)	Combined	-0.39	-1.02	0.23	0.220	0.31	0.756
Höppner et al. (2003)	Combined	0.19	-0.70	1.08	0.676	1.34	0.182
Nahas et al. (2003)	HAMD	0.09	-0.73	0.91	0.828	1.22	0.223
Buchholtz et al. (2004)	HAMD	0.14	-0.95	1.24	0.796	1.07	0.285
Holtzheimer et al. (2004)	Combined	-0.22	-1.24	0.80	0.674	0.51	0.607
Koerselman et al. (2004)	HAMD	-0.15	-0.70	0.40	0.584	0.93	0.353
Mosimann et al. (2004)	Combined	-0.12	-0.95	0.71	0.774	0.79	0.428
Poulet et al. (2004)	Combined	0.13	-0.77	1.04	0.772	1.22	0.224
Rossini et al. (2005)	HAMD	-0.71	-1.13	-0.30	0.001	-0.54	0.590
Rumi et al. (2005)	MADRS	-1.23	-1.87	-0.60	0.000	-1.66	0.098
Su et al. (2005) all	Combined	-1.14	-1.95	-0.33	0.006	-1.23	0.220
Avery et al. (2006)	Combined	-0.67	-1.16	-0.18	0.007	-0.38	0.702
Anderson et al. (2007)	MADRS	-0.78	-1.60	0.04	0.063	-0.50	0.615
Bortolomasi et al. (2007)	Combined	-1.20	-2.20	-0.19	0.020	-1.15	0.252
Herwig et al. (2007)	Combined	-0.17	-0.54	0.19	0.355	1.04	0.299
Loo et al. (2007)	Combined	-0.34	-0.98	0.30	0.298	0.42	0.673
O'Reardon et al. (2007)	Combined	-0.30	-0.53	-0.08	0.009	0.72	0.474
Stern et al. (2007)	HAMD	-2.93	-4.09	-1.77	0.000	-3.67	0.000
Bretlau et al. (2008)	HAMD	-0.75	-1.35	-0.15	0.015	-0.54	0.590
Mogg et al. (2008)	Combined	-0.46	-0.99	0.07	0.087	0.17	0.868
		-0.52	-0.69	-0.36	0.000		

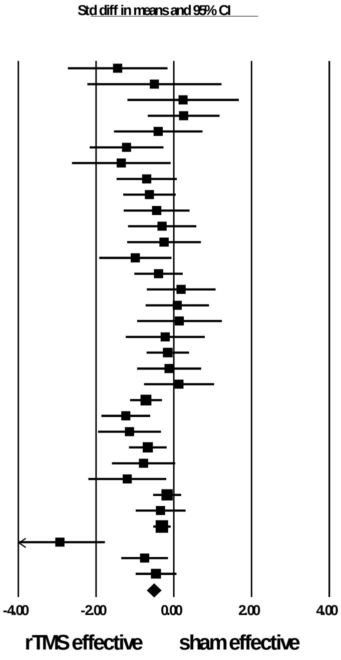

Group by Outliers	Study name	Outcome	Std diff in means	Lower limit	Upper limit	p-Value
outlier			-2.93	-4.09	-1.77	0.000
study			-0.47	-0.61	-0.33	0.000
Overall			-0.51	-0.65	-0.37	0.000

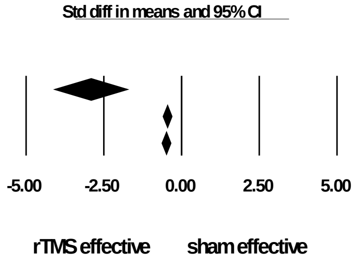

Notes: The forest plot (top) shows that one study (Stern et al. 2007) was a statistical outlier because the weighted *d* in this study (-2.93) was significantly higher (*p*<.001) than the overall mean weighted *d* of all other 32 studies (-.47; bottom plot). Thus, this study was removed from all analyses to prevent the inflation of effect sizes in the analysis of the high frequency studies. 'All' refers to all patients in multiple active rTMS groups who received rTMS with different parameters. 'Combined' in the column 'Outcome' indicates that more than one depression scale was used in a study and the effect sizes according to the multiple scales were combined into one. Abbreviations: *CI*, confidence interval; DLPFC, dorsolateral prefrontal cortex; HAMD, Hamilton Depression Rating Scale; HFL, high-frequency rTMS of the left DLPFC; MADRS, Montgomery Åsberg Depression Rating Scale; rTMS, repetitive transcranial magnetic stimulation; Std, standardised; Std diff, standardized mean difference (Cohen's *d*).

Figure S6 Cumulative random-effects meta-analysis of *N*=32 HFL studies

Study name	Outcome	Cumulative statistics: Point	Lower limit	Upper limit	p-Value	Sham	rTMS
George et al. (1997)	HAMD	-1.44	-2.73	-0.16	0.028	5	7
Avery et al. (1999)	Combined	-1.11	-2.14	-0.08	0.035	7	11
Kimbrell et al. (1999)	HAMD	-0.62	-1.65	0.41	0.240	10	16
Loo et al. (1999)	Combined	-0.31	-1.14	0.53	0.470	19	25
Padberg et al. (1999)	HAMD	-0.31	-0.93	0.32	0.336	25	31
Berman et al. (2000)	HAMD	-0.52	-1.12	0.09	0.096	35	41
Eschweiler et al. (2000)	Combined	-0.63	-1.19	-0.06	0.030	40	48
George et al. (2000) all	HAMD	-0.64	-1.09	-0.18	0.006	50	68
Garcia-Toro et al. (2001a)	Combined	-0.63	-1.00	-0.26	0.001	68	85
Garcia-Toro et al. (2001b)	Combined	-0.61	-0.93	-0.28	0.000	79	96
Manes et al. (2001)	HAMD	-0.57	-0.87	-0.28	0.000	89	106
Boutros et al. (2002)	HAMD	-0.54	-0.82	-0.26	0.000	96	117
Padberg et al. (2002)	Combined	-0.58	-0.85	-0.31	0.000	106	127
Fitzgerald et al. (2003)	Combined	-0.55	-0.80	-0.30	0.000	126	147
Höppner et al. (2003)	Combined	-0.50	-0.74	-0.26	0.000	136	157
Nahas et al. (2003)	HAMD	-0.45	-0.69	-0.22	0.000	148	168
Buchholtz et al. (2004)	HAMD	-0.43	-0.66	-0.20	0.000	155	174
Holtzheimer et al. (2004)	Combined	-0.42	-0.64	-0.20	0.000	163	181
Koerselman et al. (2004)	HAMD	-0.38	-0.58	-0.18	0.000	188	207
Mosimann et al. (2004)	Combined	-0.37	-0.56	-0.17	0.000	197	222
Poulet et al. (2004)	Combined	-0.34	-0.54	-0.15	0.000	206	232
Rossini et al. (2005)	HAMD	-0.41	-0.59	-0.23	0.000	253	281
Rumi et al. (2005)	MADRS	-0.45	-0.65	-0.25	0.000	277	303
Su et al. (2005) all	Combined	-0.48	-0.68	-0.28	0.000	287	323
Avery et al. (2006)	Combined	-0.50	-0.68	-0.31	0.000	320	358
Anderson et al. (2007)	MADRS	-0.51	-0.69	-0.33	0.000	334	369
Bortolomasi et al. (2007)	Combined	-0.53	-0.70	-0.35	0.000	341	381
Herwig et al. (2007)	Combined	-0.49	-0.66	-0.32	0.000	400	438
Loo et al. (2007)	Combined	-0.49	-0.65	-0.32	0.000	419	457
O'Reardon et al. (2007)	Combined	-0.46	-0.61	-0.31	0.000	565	612
Bretlau et al. (2008)	HAMD	-0.48	-0.62	-0.33	0.000	588	634
Mogg et al. (2008)	Combined	-0.47	-0.61	-0.33	0.000	617	662
		-0.47	-0.61	-0.33	0.000		

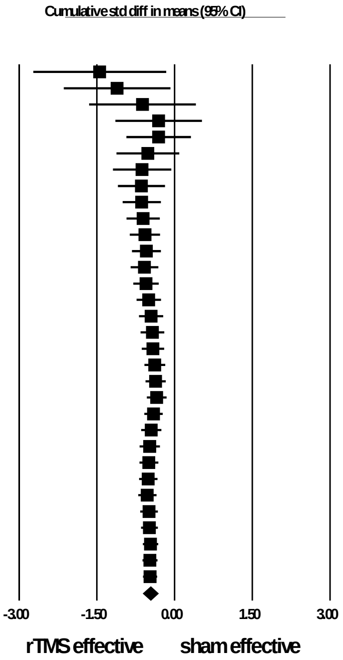

Notes: 'All' refers to all patients in multiple active rTMS groups who received rTMS with different parameters. 'Combined' in the column 'Outcome' indicates that more than one depression scale was used in a study and the effect sizes according to the multiple scales were combined into one. 'Point' refers to the overall mean weighted *d* of all studies before and including the study listed in each row. Abbreviations: *CI*, confidence interval; DLPFC, dorsolateral prefrontal cortex; HAMD, Hamilton Depression Rating Scale; HFL, high-frequency rTMS of the left DLPFC; MADRS, Montgomery Åsberg Depression Rating Scale; rTMS, repetitive transcranial magnetic stimulation; Std diff, standardized mean difference (Cohen's *d*).

Figure S7 One-study removed random-effects meta-analysis of *N*=32 HFL studies

Studyname	Outcome	Statistics with studyremoved			
		Point	Lower limit	Upper limit	p-Value
George et al. (1997)	HAMD	-0.46	-0.60	-0.32	0.000
Avery et al. (1999)	Combined	-0.47	-0.62	-0.33	0.000
Kimbrell et al. (1999)	HAMD	-0.48	-0.62	-0.34	0.000
Loo et al. (1999)	Combined	-0.49	-0.62	-0.35	0.000
Padberg et al. (1999)	HAMD	-0.47	-0.62	-0.33	0.000
Berman et al. (2000)	HAMD	-0.46	-0.59	-0.32	0.000
Eschweiler et al. (2000)	Combined	-0.46	-0.60	-0.32	0.000
George et al. (2000) all	HAMD	-0.47	-0.61	-0.32	0.000
Garcia-Toro et al. (2001a)	Combined	-0.47	-0.61	-0.32	0.000
Garcia-Toro et al. (2001b)	Combined	-0.47	-0.62	-0.33	0.000
Manes et al. (2001)	HAMD	-0.48	-0.62	-0.33	0.000
Boutros et al. (2002)	HAMD	-0.48	-0.62	-0.34	0.000
Padberg et al. (2002)	Combined	-0.46	-0.60	-0.32	0.000
Fitzgerald et al. (2003)	Combined	-0.48	-0.62	-0.33	0.000
Höppner et al. (2003)	Combined	-0.49	-0.62	-0.35	0.000
Nahas et al. (2003)	HAMD	-0.49	-0.63	-0.35	0.000
Buchholtz et al. (2004)	HAMD	-0.48	-0.62	-0.34	0.000
Holtzheimer et al. (2004)	Combined	-0.48	-0.62	-0.34	0.000
Koerselman et al. (2004)	HAMD	-0.49	-0.63	-0.35	0.000
Mosimann et al. (2004)	Combined	-0.48	-0.62	-0.34	0.000
Poulet et al. (2004)	Combined	-0.48	-0.62	-0.34	0.000
Rossini et al. (2005)	HAMD	-0.45	-0.60	-0.31	0.000
Rumi et al. (2005)	MADRS	-0.43	-0.56	-0.31	0.000
Su et al. (2005) all	Combined	-0.45	-0.59	-0.32	0.000
Avery et al. (2006)	Combined	-0.46	-0.61	-0.32	0.000
Anderson et al. (2007)	MADRS	-0.47	-0.61	-0.32	0.000
Bortolomasi et al. (2007)	Combined	-0.46	-0.60	-0.32	0.000
Herwig et al. (2007)	Combined	-0.50	-0.64	-0.35	0.000
Loo et al. (2007)	Combined	-0.48	-0.62	-0.33	0.000
O'Reardon et al. (2007)	Combined	-0.50	-0.65	-0.34	0.000
Bretlau et al. (2008)	HAMD	-0.46	-0.60	-0.32	0.000
Mogg et al. (2008)	Combined	-0.48	-0.62	-0.33	0.000
		-0.47	-0.61	-0.33	0.000

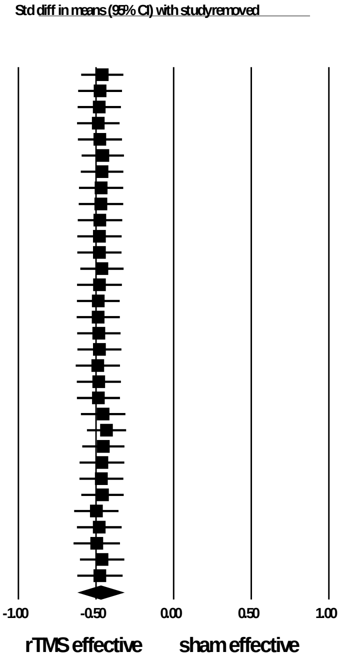

Notes: ‘All’ refers to all patients in multiple active rTMS groups who received rTMS with different parameters. ‘Combined’ in the column ‘Outcome’ indicates that more than one depression scale was used in a study and the effect sizes according to the multiple scales were combined into one. ‘Point’ refers to the overall mean weighted *d* of all studies except the study listed in each row. Abbreviations: *CI*, confidence interval; DLPFC, dorsolateral prefrontal cortex; HAMD, Hamilton Depression Rating Scale; HFL, high-frequency rTMS of the left DLPFC; MADRS, Montgomery Åsberg Depression Rating Scale; rTMS, repetitive transcranial magnetic stimulation; Std diff, standardized mean difference (Cohen’s *d*).

Figure S8 Meta-regression of % female on weighted *d* in *N*=31 HFL studies

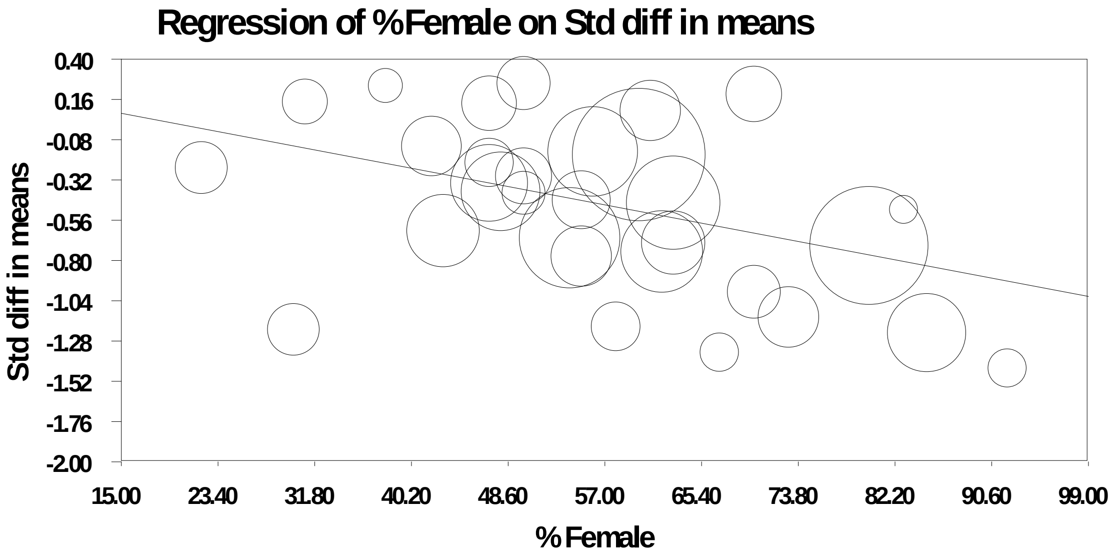

Notes: The figure shows a scatterplot of weighted *d*/study (Y-axis) versus proportion of female patients/study (X-axis) according to a univariate meta-regression of 31 high-frequency studies. The removal of the study with the largest weight (O'Reardon et al. 2007) did not change the outcome of this analysis (*B*=-.01, slope $p_{two\text{-}tailed}$=.006). Thus, depression scores were significantly reduced after high-frequency rTMS compared to sham in studies with higher proportion of female patients after exclusion of the largest study from the analysis. Abbreviations: DLPFC, dorsolateral prefrontal cortex; HFL, high-frequency rTMS of the left DLPFC; rTMS, repetitive transcranial magnetic stimulation; Std diff, standardized mean difference (Cohen's *d*).

References

Anderson, I, Delvai, N, Ashim, B, Ashim, S, Lewin, C, Singh, V, et al. (2007). Adjunctive fast repetitive transcranial magnetic stimulation in depression. *British Journal of Psychiatry, 190*, 533-534.

Avery, D, Claypoole, K, Robinson, L, Neumaier, J, Dunner, D, Scheele, L, et al. (1999). Repetitive transcranial magnetic stimulation in the treatment of medication-resistant depression: preliminary data. *Journal of Nervous and Mental Disease*, *187*(2), 114-117.

Avery, D, Holtzheimer III, P, Fawaz, W, Russo, J, Haynor, D, Claypoole, K, et al. (2006). A controlled study of repetitive transcranial magnetic stimulation in medication-resistant major depression. *Biological Psychiatry, 59*, 187-194.

Avery, D, Neumaier, J, Roy-Byrne, P, Dunner, D, Claypoole, K, & Tucker, G (2000). *TMS in the treatment of medication-free major depression (abstract).* Paper presented at the International Society for Transcranial Stimulation Satellite Meeting at the Society of Biological Psychiatry Annual Meeting (May 10, 2000), Chicago, USA,

Berman, R, Hoffman, R, Narashima, M, Hu, X, Sanacora, G, Charney, D, et al. (2000). A randomized clinical trial of repetitive transcranial magnetic stimulation in the treatment of major depression. *Biological Psychiatry, 47*, 332-337.

Borenstein, M, Hedges, L, Higgins, J, & Rothstein, H (2009). *Introduction to meta-analysis*. Great Britain: John Wiley & Sons, Ltd.

Bortolomasi, M, Minelli, A, Fuggetta, G, Perini, M, Comencini, S, Fiaschi, A, et al. (2007). Long-lasting effects of high frequency repetitive transcranial magnetic stimulation in major depressed patients. *Psychiatry Research, 150*(2), 181-186.

Boutros, NN, Gueorguieva, R, Hoffman, RE, Oren, DA, Feingold, A, & Berman, RM (2002). Lack of a therapeutic effect of a 2-week sub-threshold transcranial magnetic stimulation course for treatment-resistant depression. *Psychiatry Research, 113*(3), 245-254, doi:10.1016/s0165-1781(02)00267-6.

Bretlau, L, Lindberg, L, Unden, M, Dissing, S, & Bech, P (2008). Repetitive transcranial magnetic stimulation (rTMS) in combination with escitalopram in patients with treatment-resistant major depression.A double-blind,randomised, sham-controlled trial. *Pharmacopsychiatry, 41*(2), 41-47.

Buchholtz, H, Videbech, P, Clemmensen, K, Sturlason, R, Jensen, H, & Vestergaard, P (2004). Repetitive transcranial magnetic stimulation as add-on antidepressant treatment. The applicability of the method in a clinical setting. *Nordic Journal of Psychiatry, 58*(6), 455-457.

Burt, T, Lisanby, S, & Sackeim, H (2002). Neuropsychiatric applications of transcranial magnetic stimulation: a meta analysis. *International Journal of Neuropsychopharmacology, 5*(1), 73-103.

Conca, A, Koppi, S, Konig, P, Swoboda, E, & Krecke, N (1996). Transcranial magnetic stimulation: a novel antidepressive strategy? *Neuropsychobiology, 34*(4), 204-207.

Couturier, J (2005). Efficacy of rapid-rate repetitive transcranial magnetic stimulation in the treatment of depression: a systematic review and meta-analysis. *Journal of Psychiatry & Neuroscience, 30*(2), 83-90.

Dell'Osso, B, Camuri, G, Castellano, F, Vecchi, V, Benedetti, M, Bortolussi, S, et al. (2011). Meta-review of metanalytic studies with repetitive transcranial magnetic stimulation (rTMS) for the treatment of major depression. *Clinical Practice and Epidemiology in Mental Health, 7*, 167-177.

DerSimonian, R, & Laird, N (1986). Meta-analysis in clinical trials. *Controlled Clinical Trials, 7*, 177-188.

Eschweiler, G, Wegerer, C, Schlotter, W, Spandl, C, Stevens, A, Bartels, M, et al. (2000). Left prefrontal activation predicts therapeutic effects of repetitive transcranial magnetic stimulation (rTMS) in major depression. *Psychiatry Research: Neuroimaging, 99*, 161-172.

Fitzgerald, P, Benitez, J, de Castella, A, Daskalakis, Z, Brown, T, & Kulkarni, J (2006). A randomized, controlled trial of sequestial bilateral repetitive transcranial magnetic stimulation for treatment-resistant depression. *American Journal of Psychiatry, 163*, 88-94.

Fitzgerald, P, Brown, T, Marston, N, Daskalakis, Z, de Castella, A, & Kulkarni, J (2003). Transcranial magnetic stimulation in the treatment of depression: a double-blind, placebo-controlled trial. *Archives of General Psychiatry, 60*, 1002-1008.

Fitzgerald, P, Hoy, K, McQueen, S, Herring, S, Segrave, R, Been, G, et al. (2008). Priming stimulation enhances the effectiveness of low-frequency right prefrontal cortex transcranial magnetic stimulation in major depression. *Journal Of Clinical Psychopharmacology, 28*(1), 52-58.

Garcia-Toro, M, Mayol, A, Arnillas, H, Capllonch, I, Ibarra, O, Crespí, M, et al. (2001). Modest adjunctive benefit with transcranial magnetic stimulation in medication-resistant depression. *Journal of Affective Disorders, 64*(2-3), 271-275.

García-Toro, M, Pascual-Leone, A, Romera, M, González, A, Micó, J, Ibarra, O, et al. (2001). Prefrontal repetitive transcranial magnetic stimulation as add on treatment in depression. *Journal of Neurology, Neurosurgery and Psychiatry, 71*, 546-548.

Garcia-Toro, M, Salva, J, Daumal, J, Andres, J, Romera, M, Lafaub, O, et al. (2006). High (20-Hz) and low (1-Hz) frequency transcranial magnetic stimulation as adjuvant treatment in medication-resistant depression. *Psychiatry Research: Neuroimaging, 146*, 53-57.

George, M, Nahas, Z, Molloy, M, Speer, A, Oliver, N, Li, X, et al. (2000). A controlled trial of daily left prefrontal cortex TMS for treating depression. *Biological Psychiatry, 48*, 962-970.

George, M, Wassermann, EM, Williams, WE, Kimbrell, TA, Little, JT, Hallett, M, et al. (1997). Mood improvement following daily left prefrontal repetitive transcranial magnetic stimulation in patients with depression: A placebo-controlled crossover trial. *American Journal of Psychiatry, 154,* 1752-1756.

Gross, M, Nakamura, L, Pascual-Leone, A, & Fregni, F (2007). Has repetitive transcranial magnetic stimulation (rTMS) treatment for depression improved? A systematic review and meta-analysis comparing the recent vs. the earlier rTMS studies. *Acta Psychiatrica Scandinavica, 116*, 165-173.

Grunhaus, L, Dannon, P, Schreiber, S, Dolberg, O, Amiaz, R, Ziv, R, et al. (2000). Repetitive transcranial magnetic stimulation is as effective as electroconvulsive therapy in the treatment of nondelusional major depressive disorder: an open study. *Biological Psychiatry, 47*(4), 314-324.

Haag, C, Padberg, F, Thoma, H, Zwanzger, P, Hampel, H, & Moller, H (1997). Rapid transcranial magnetic stimulation (rTMS) in the treatment of major depression: a randomised placebo controlled study (abstract). *Pharmacopsychiatry, 30,* 173.

Hausmann, A, Kemmler, G, Walpoth, M, Mechtcheriakov, S, Kramer-Reinstadler, K, Lechner, T, et al. (2004). No benefit derived from repetitive transcranial magnetic stimulation in depression: a prospective, single-centre, randomised, double blind, sham-controlled "add on" trial. *Journal of Neurology, Neurosurgery and Psychiatry, 75*(2), 320-322.

Herrmann, L, & Ebmeier, K (2006). Factors modifying the efficacy of transcranial magnetic stimulation in the treatment of depression: A Review. *Journal of Clinical Psychiatry, 67*(12), 1870-1876.

Herwig, U, Fallgatter, A, Hoppner, J, Eschweiler, G, Kron, M, Hajak, G, et al. (2007). Antidepressant effects of augmentative transcranial magnetic stimulation. Randomised multicentre trial. *British Journal of Psychiatry, 191,* 441-448.

Herwig, U, Lampe, Y, Juengling, F, Wunderlich, A, Walter, H, Spitzer, M, et al. (2003). Add-on rTMS for treatment of depression: a pilot study using stereotaxic coil-navigation according to PET data. *Journal Of Psychiatric Research, 37*(4), 267-275.

Holtzheimer, P, Russo, J, Claypoole, K, Roy-Byrne, P, & Avery, D (2004). Shorter duration of depressive episode may predict response to repetitive transcranial magnetic stimulation. *Depression and Anxiety, 19*, 24-30.

Holtzheimer, Pr, Russo, J, & Avery, D (2001). A meta-analysis of repetitive transcranial magnetic stimulation in the treatment of depression. *Psychopharmacological Bulletin, 35*(4), 149-169.

Höppner, J, Schulz, M, Irmisch, G, Mau, R, Schläfke, D, & Richter, J (2003). Antidepressant efficacy of two different rTMS procedures:High frequency over left versus low frequency over right prefrontal cortex compared with sham stimulation. *European Archives Of Psychiatry And Clinical Neuroscience, 253*, 103-109.

Januel, D, Dumortier, G, Verdon, C, Stamatiadis, L, Saba, G, Cabaret, W, et al. (2006). A double-blind sham controlled study of right prefrontal repetitive transcranial magnetic stimulation (rTMS): Therapeutic and cognitive effect in medication free unipolar depression during 4 weeks. *Progress In Neuro-Psychopharmacology & Biological Psychiatry, 30*, 126-130.

Jorge, R, Robinson, R, Tateno, A, Narushima, K, Acion, L, Moser, D, et al. (2004). Repetitive transcranial magnetic stimulation as treatment of poststroke depression: A preliminary study. *Biological Psychiatry, 55*, 398-405.

Kauffmann, C, Cheema, M, & Miller, B (2004). Slow right prefrontal transcranial magnetic stimulation as a treatment for medication-resistant depression: A double-blind, placebo-controlled study. *Depression and Anxiety, 19*, 59-62.

Kedzior, K, Azorina, V, & Reitz, S (2014). More female patients and fewer stimuli per session are associated with the short-term antidepressant properties of repetitive transcranial magnetic stimulation (rTMS): a meta-analysis of 54 sham-controlled studies published between 1997–2013. *Neuropsychiatric Disease and Treatment, 10,* 727-756.

Kimbrell, T, Little, J, Dunn, R, Frye, M, Greenberg, B, Wassermann, E, et al. (1999). Frequency dependence of antidepressant response to left prefrontal repetitive transcranial magnetic stimulation (rTMS) as a function of baseline cerebral glucose metabolism. *Biological Psychiatry, 46*, 1603-1613.

Klein, E, Kreinin, I, & Chistyakov, A (1999). Therapeutic efficacy of right prefrontal slow repetitive transcranial magnetic stimulation in major depression: A double blind controlled study. *Archives of General Psychiatry, 56,* 315-320.

Koerselman, F, Laman, D, van Duijn, H, van Duijn, M, & Willems, M (2004). A 3-month, follow-up, randomized, placebo-controlled study of repetitive transcranial magnetic stimulation in depression. *Journal of Clinical Psychiatry, 65,* 1323-1328.

Kolbinger, H, Hoflich, G, Hufnagel, A, Moller, H, & Kasper, S (1995). Transcranial magnetic stimulation (TMS) in the treatment of major depression-a pilot study. *Human Psychopharmacology, 10*, 305-310.

Kozel, F, & George, M (2002). Meta-analysis of left prefrontal repetitive transcranial magnetic stimulation (rTMS) to treat depression. *Journal of Psychiatric Practice, 8*(5), 270-275.

Lam, R, Chan, P, Wilkins-Ho, M, & Yatham, L (2008). Repetitive transcranial magnetic stimulation for treatment-resistant depression: A systematic review and meta-ananlysis. *Canadian Journal Of Psychiatry. Revue Canadienne De Psychiatrie, 53*(9), 621-631.

Lisanby, SH, Pascual-Leone, A, Sampson, S, Boylan, L, Burt, T, & Sackeim, H (2001). Augmentation of sertraline antidepressant treatment with transcranial magnetic stimulation (abstract). *Biological Psychiatry, 49,* 81S.

Loo, C, Mitchell, P, Croker, V, Malhi, G, Wen, W, Gandevia, S, et al. (2003). Double-blind controlled investigation of bilateral prefrontal transcranial magnetic stimulation for the treatment of resistant major depression. *Psychological Medicine, 33,* 33-40.

Loo, C, Mitchell, P, McFarquhar, T, Malhi, G, & Sachdev, P (2007). A sham-controlled trial of the efficacy and safety of twice-daily rTMS in major depression. *Psychological Medicine, 37*, 341-349.

Loo, C, Mitchell, P, Sachdev, P, McDarmont, B, Parker, G, & Gandevia, S (1999). Double-blind controlled investigation of transcranial magnetic stimulation for the treatment of resistant major depression. *American Journal of Psychiatry, 156*, 946-948.

Manes, F, Jorge, R, Morcuende, M, Yamada, T, Paradiso, S, & Robinson, R (2001). A controlled study of repetitive transcranial magnetic stimulation as a treatment of depression in the elderly. *International Psychogeriatrics, 13*(2), 225-231.

Martin, J, Barbanoj, M, Schlaepfer, T, Thompson, E, Perez, V, & Kulisevsky, J (2003). Repetitive transcranial magnetic stimulation for the treatment of depression. Systematic review and meta-analysis. *British Journal of Psychiatry, 182*, 480-491.

McNamara, B, Ray, JL, Arthurs, OJ, & Boniface, S (2001). Transcranial magnetic stimulation for depression and other psychiatric disorders. *Psychological Medicine, 31*(7), 1141-1146.

Miniussi, C, Bonato, C, Bignotti, S, Gazzoli, A, Gennarelli, M, Pasqualetti, P, et al. (2005). Repetitive transcranial magnetic stimulation (rTMS) at high and low frequency: an efficacious therapy for major drug-resistant depression? *Clinical Neurophysiology, 116*, 1062-1071.

Mogg, A, Pluck, G, Eranti, S, Landau, S, Purvis, R, Brown, R, et al. (2008). A randomized controlled trial with 4-month follow-up of adjunctive repetitive transcranial magnetic stimulation of the left prefrontal cortex for depression. *Psychological Medicine, 38*, 323-333.

Moher, D, Liberati, A, Tetzlaff, J, & Altman, D (2009). Preferred reporting items for systematic reviews and meta-analyses: the PRISMA statement. *British Medical Journal, 339*, b2535.

Moller, A, Hjaltason, O, Ivarsson, O, & Stefansson, S (2006). The effects of repetitive transcranial magnetic stimulation on depressive symptoms and the P300 event-related potential. *Nordic Journal of Psychiatry, 60*(4), 282-285.

Mosimann, U, Schmitt, W, Greenberg, B, Kosel, M, Muri, R, Berkhoff, M, et al. (2004). Repetitive transcranial magnetic stimulation: a putative add-on treatment for major depression in elderly patients. *Psychiatry Research, 126*, 123-133.

Nahas, Z, Kozel, F, Li, X, Anderson, B, & George, M (2003). Left prefrontal transcranial magnetic stimulation (rTMS) treatment of depression in bipolar affective disorder:a pilot study of acute safety and efficacy. *Bipolar Disorders, 5*(1), 40-47.

O'Reardon, J, Solvason, H, Janicak, P, Sampson, S, Isenberg, K, Nahas, Z, et al. (2007). Efficacy and safety of transcranial magnetic stimulation in the acute treatment of major depression: A multisite randomized controlled trial. *Biological Psychiatry, 62,* 1208-1216.

Padberg, F, Zwanzger, P, Keck, M, Kathmann, N, Mikhaiel, P, Ella, R, et al. (2002). Repetitive transcranial magnetic stimulation (rTMS) in major depression: relation between efficacy and stimulation intensity. *Neuropsychopharmacology, 27*(4), 638-645.

Padberg, F, Zwanzger, P, Thoma, H, Kathmann, N, Haag, C, Greenberg, B, et al. (1999). Repetitive transcranial magnetic stimulation (rTMS) in pharmacotherapy-refractory major depression: comparative study of fast, slow and sham rTMS. *Psychiatry Research, 88,* 163-171.

Pascual-Leone, A, Rubio, B, Pallardo, F, & Catala, MD (1996). Rapid-rate transcranial magnetic stimulation of the left dorsolateral prefrontal cortex in drug-resistant depression. *Lancet, 348,* 233-237.

Poulet, E, Brunelin, J, Boeuve, C, Lerond, J, Amato, T, Dalery, J, et al. (2004). Repetitive transcranial magnetic stimulation does not potentiate antidepressant treatment. *European Psychiatry, 19,* 382-383.

Rodriguez-Martin, J, Barbanoj, M, Schlaepfer, T, Clos, S, Perez, V, Kulisevsky, J, et al. (2001). Transcranial magnetic stimulation for treating depression. *Cochrane Database of Systematic Reviews, 4,* CD003493.

Rossini, D, Lucca, A, Zanardi, R, Magri, L, & Smeraldi, E (2005). Transcranial magnetic stimulation in treatment-resistant depressed patients: A double-blind, placebo-controlled trial. *Psychiatry Research, 137,* 1-10.

Rossini, D, Magri, L, Lucca, A, Giordani, S, Smeraldi, E, & Zanardi, R (2005). Does rTMS hasten the response to escitalopram, sertraline, or venlafaxine in patients with major depressive disorder? A double-blind, randomized, sham-controlled trial. *Journal of Clinical Psychiatry, 66*(12), 1569-1575.

Rumi, D, Gattaz, W, Rigonatti, S, Rosa, M, Fregni, F, Rosa, M, et al. (2005). Transcranial magnetic stimulation accelerates the antidepressant effect of amitriptyline in severe depression: a double-blind placebo-controlled study. *Biological Psychiatry, 57*, 162-166.

Schutter, D (2009). Antidepressant efficacy of high-frequency transcranial magnetic stimulation over the left dorsolateral prefrontal cortex in double-blind sham-controlled designs: a meta-analysis. *Psychological Medicine, 39*, 65-75.

Schutter, D (2010). Quantitative review of the efficacy of slow-frequency magnetic brain stimulation in major depressive disorder. *Psychological Medicine, 40*(11), 1789-1795.

Slotema, C, Blom, J, Hoek, H, & Sommer, I (2010). Should we expand the toolbox of psychiatric treatment methods to include Repetitive Transcranial Magnetic Stimulation (rTMS)? A meta-analysis of the efficacy of rTMS in psychiatric disorders. *Journal of Clinical Psychiatry, 71*(7), 873-884.

Stern, W, Tormos, J, Press, D, Pearlman, C, & Pascual-Leone, A (2007). Antidepressant effects of high and low frequency repetitive transcranial magnetic stimulation to the dorsolateral prefrontal cortex:a double-blind, randomized, placebo- controlled trial. *Journal of Neuropsychiatry and Clinical Neurosciences, 19*(2), 179-186.

Stikhina, N, Lyskov, E, Lomarev, M, Aleksanian, Z, Mikhaĭlov, V, & Medvedev, S (1999). [Transcranial magnetic stimulation in neurotic depression]. *Zhurnal Nevropatologii i Psikhiatrii Imieni SS Korsakova, 99*(10), 26-29.

Su, T, Huang, C, & Wei, I (2005). Add-on rTMS for medication-resistant depression:a randomized, double-blind,sham-controlled trial in Chinese patients. *Journal of Clinical Psychiatry, 66*(7), 930-937.

Szuba, M, O'Reardon, J, Rai, A, Snyder-Kastenberg, J, Amsterdam, J, Gettes, D, et al. (2001). Acute mood and thyroid stimulating hormone effects of transcranial magnetic stimulation in major depression. *Biological Psychiatry, 50*, 22-27.
